# The Paradox of E-Cadherin: Role in response to hypoxia in the tumor microenvironment and regulation of energy metabolism

**DOI:** 10.18632/oncotarget.872

**Published:** 2013-03-21

**Authors:** Khoi Chu, Kimberley M. Boley, Ricardo Moraes, Sanford H Barsky, Fredika M. Robertson

**Affiliations:** ^1^ Department of Experimental Therapeutics, The University of Texas MD Anderson Cancer Center, Houston, TX; ^2^ Department of Pathology, The University of Nevada School of Medicine, Reno, NV

**Keywords:** E-Cadherin, Inflammatory Breast Cancer, Mesenchymal-Epithelial Transition (MET), Hypoxia-inducible 1α transcription factor (HIF-1α), Metabolism, Glycolysis

## Abstract

E-Cadherin is a cell:cell adhesion molecule critical for appropriate embryonic and mammary development. In cancer, E-Cadherin has been primarily viewed as being lost during the process of epithelial-mesenchymal transition (EMT), which occurs with a switch from E-Cadherin expression to a gain of N-Cadherin and other mesenchymal markers. EMT has been shown to play a role in the metastatic process while the reverse process, mesenchymal-epithelial transition (MET), is important for metastatic colonization. Here we report an unexpected role of E-Cadherin in regulating tumorigenicity and hypoxia responses of breast tumors *in vivo*. Reduced expression of E-Cadherin led to a dramatic reduction of the *in vivo* growth capability of SUM149, Mary-X and 4T1 tumor cells. Furthermore, over-expression of *ZEB1*, a known transcriptional repressor of E-Cadherin, led to reduced *in vivo* growth of SUM149 tumors. Gene set enrichment analysis identified the loss of hypoxia response genes as a major mechanism in mediating the lack of *in vivo* growth of SUM149 cells that lacked E-Cadherin or over-expressed *ZEB1*. The *in vivo* growth defect of SUM149 E-Cadherin knockdown tumors was rescued by the hypoxia-inducible 1α transcription factor (HIF-1α). Given the importance of HIF-1α in cellular metabolism, we observed reduced glycolytic capacity in SUM149 and 4T1 cells that had E-Cadherin knocked down. Our observations shed light on the complex functions of E-Cadherin in retention of an epithelial phenotype and as a mediator of survival of aggressive breast cancer under hypoxic conditions *in vivo*. Furthermore, we find that patients with basal subtype breast cancer and high E-Cadherin expression in their tumors had a poor clinical outcome. Our data suggests a novel function for E-Cadherin as a bona fide signaling molecule required for the *in vivo* growth of aggressive breast cancer tumor cells, that retain E-Cadherin expression, in mediating their metabolic function.

## INTRODUCTION

E-Cadherin is a glycoprotein involved in cell:cell adhesion and is essential for appropriate embryonic and mammary development [1-3]. In recent years, paradoxical roles for E-Cadherin in tumor progression have been described in numerous types of cancer. While the current paradigm of the acquisition of an invasive phenotype has been associated with a loss of E-Cadherin during the process of the epithelial mesenchymal transition (EMT), studies in a number of carcinoma types show that tumors are quite heterogeneous and loss of E-Cadherin is not always associated with increased invasive behavior. Similarly maintenance of E-Cadherin expression is not a detrimental to invasion and metastasis.

While there has been a focus on the process of EMT, recent studies have begun to appreciate that the maintenance or re-acquisition of an epithelial phenotype as well as the reversion to a MET phenotype, defined by the expression of E-Cadherin, as a requirement for tumor colonization at sites distant from the primary tumor [4, 5]. Maintenance of E-Cadherin expression in highly metastatic cell lines has been described in a number of pre-clinical models of metastasis of breast (4T1), prostate (DU145), and bladder (TSU-Pr1) cancers [6-8]. This has recently been referred to as “the dark side of E-Cadherin” [9, 10]. E-Cadherin has been reported to be frequently expressed in metastatic foci in both clinical samples [4, 5] and experimental models of breast cancer metastasis [11]. Numerous studies have now documented a role for E-Cadherin in metastasis and tumor colonization. As an example, in ovarian carcinoma E-Cadherin expression is maintained and no EMT occurs, instead a state of mesenchymal-epithelial transition (MET) is observed [12, 13]. Inflammatory breast cancer (IBC) is an aggressive form of locally advanced breast cancer where local lymph node are involved and is phenotypically distinct from other variants of this disease (reviewed in [14]). In IBC, despite its aggressiveness, E-Cadherin expression is maintained in the primary tumor and tumor emboli. Thus, IBC represents the prototype breast cancer with prominent MET similar to ovarian cancer.

Collectively, these studies suggest that E-Cadherin serves a function beyond its role in cell:cell adhesion. The cellular plasticity observed during the reversible processes of EMT and MET suggest that cancer cells retain tight control in the degree of cellular re-programming and may select EMT features such as invasion while simultaneously maintaining epithelial features such as E-Cadherin expression. Indeed, *in vitro* studies in breast cancer cell lines suggested that E-Cadherin expression is insufficient to block invasion [15].

Using IBC as a prototype pre-clinical model for elucidating the role of MET in aggressive cancer, we manipulated the levels of E-Cadherin via shRNA knockdown and over-expression of *ZEB1*, a known transcriptional repressor of E-Cadherin. The present studies demonstrate that E-Cadherin is required for *in vivo* growth of SUM149 and Mary-X cells derived from IBC patients and *in vivo* growth of mammary carcinoma 4T1 tumors. The requirement for E-Cadherin for the *in vivo* growth of SUM149 tumors was found to be related to the expression of genes involved in the hypoxic response, identifying a previously unrecognized signaling function for E-Cadherin in regulating the response of tumor cells to the microenvironment. Furthermore, the *in vivo* growth defect in E-Cadherin knockdown SUM149 cells was overcome by inducing over-expression of HIF-1α. Given the importance in HIF-1α in regulating glucose metabolism, we show reduced glycolysis and L-lactate production in SUM149 and 4T1 cells with E-Cadherin knockdown. The results presented here provide a novel function for E-Cadherin in aggressive breast cancer that retain E-Cadherin expression.

## RESULTS

### E-Cadherin is associated with poor prognosis in breast cancer

**Figure 1:**
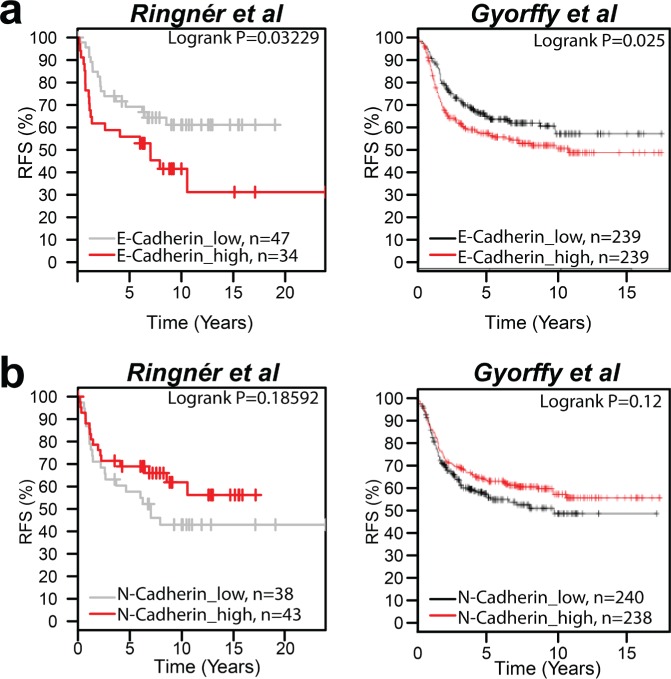
E-Cadherin but not N-Cadherin expression correlates to a poor clinical outcome in patients with breast cancer (a) Kaplan-Meier curve depicting the correlation between E-Cadherin (201131_s_at) expression and relapse-free survival (RFS) in primary basal breast tumors using two public database of breast cancer microarrays [16, 17]. (b) Kaplan-Meier curve of N-Cadherin (203441_s_at) expression and RFS in primary basal breast tumors. Multivariate analysis with estrogen receptor-negative and PAM50 basal identification was used for the Ringner et al dataset [17]. Univariate analysis was performed for the Gyorffy et al dataset [16]. Logrank P values are indicated.

To determine whether E-Cadherin expression correlates with prognosis in patients with basal breast cancer, we analyzed 2 public breast cancer databases which had outcome data [16, 17]. Patients were segregated into those with E-Cadherin expression above the mean, which were considered high expressors, and those below the mean, which were designated as low expressors. High expression of E-Cadherin was associated with poor regression free survival (RFS) (*P*=0.03229 and *P*=0.025; Fig. 1a) while N-Cadherin expression was not significantly associated with RFS (*P*=0.18592 and P=0.12; Fig. 1b) in either database. The poor RFS associated with E-Cadherin expression in breast cancer was observed in unselected tumors, as well as in breast tumors that were within both the luminal A subtype and HER2 subtype (Sup. Fig.1).

The positive association been poor prognosis and E-Cadherin expression is consistent with reports of this association in both ovarian cancer and IBC as well as in numerous models of metastatic cancers [6-8, 12-14]. To explore the relevance of this observation, we performed studies in which the levels of E-Cadherin were manipulated in E-Cadherin expressing breast cancer cell lines with an emphasis on the very aggressive IBC variant of locally advanced breast cancer.

### Expression of epithelial mesenchymal transition markers in IBC and non-IBC cell lines

**Figure 2:**
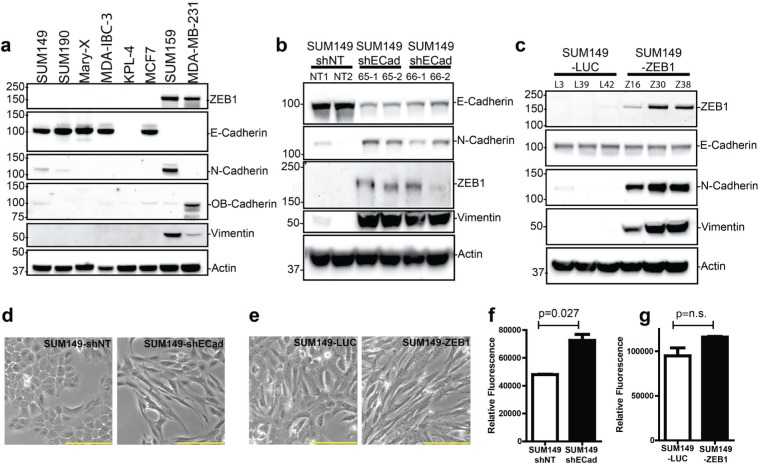
Expression of proteins associated with the EMT process including E-Cadherin and ZEB1 in IBC and non-IBC cell lines (a) IBC cell lines express E-Cadherin protein. The majority of IBC cell lines, including SUM149, SUM190, MDA-IBC-3, Mary-X express E-Cadherin, while the triple negative cell lines SUM159 and MDA-MD-231 expressed the mesenchymal cadherins, N-Cadherin and OB-Cadherin, respectively, and lack E-Cadherin protein expression. (b). Knockdown of E-Cadherin in SUM149 cells. The SUM149 cells were transduced with non-targeted shRNA (shNT) or 2 independent shRNAs against E-Cadherin (shECad-65 and shECad-66). For each construct, 2 single clones were selected and expanded. (c) Overexpression of ZEB1 in SUM149 cells. The SUM149 cells were transduced with control vector (Luciferase) or ZEB1 cDNA. For each construct, 3 single clones were selected and expanded. (d) Morphological changes were observed in SUM149 E-Cadherin knock-down clones. Reduced expression of E-Cadherin led to a more elongated mesenchymal cell morphology. Shown are representative images. Bar= 100 μM. (e). ZEB1 expression led to morphological changes including a more elongated cell morphology. Bar= 100 μM. (f). Knockdown of E-Cadherin in SUM149 led to increase Matrigel invasion assay (p<0.05). (g). Lack of change of Matrigel invasion was observed SUM149 overexpressing ZEB1 (p=0.142).

We screened a panel of IBC and non-IBC cell lines for their expression of epithelial and mesenchymal markers. The established IBC cell lines SUM149, Mary-X, SUM190, and MDA-IBC-3 express E-Cadherin protein but lack expression of other cadherin proteins such as N-Cadherin and OB-Cadherin (Fig. 2a). E-Cadherin was also expressed in 2 novel IBC xenograft models, FC-IBC01 and FC-IBC02 that we recently developed from tumor cells derived from malignant pleural effusion of consented IBC patients (Sup. Fig. S2). The IBC cell lines lacked expression of the zinc finger E-box binding homeobox 1 (ZEB1), a nuclear transcription factor that represses E-Cadherin expression. Conversely, E-Cadherin protein was not expressed in the MDA-MB-231 and SUM159 cell lines that have been defined as triple negative breast cancer cells with mesenchymal properties [18], while these breast tumor cells lines did express mesenchymal cadherins, either OB-cadherin or N-Cadherin respectively, in addition to expression of ZEB by both cell lines. Expression of an additional marker of the EMT process, vimentin, was restricted to the basal cell lines SUM159 and MDA-MB-231. Thus, E-Cadherin represents a consistent marker in IBC cell lines.

### Knockdown of E-Cadherin in SUM149 leads to induction of EMT markers

To further evaluate the role of E-Cadherin in breast tumor growth and metastasis, two lentiviral based shRNA targeting the *CDH1* gene were used to generated stable E-Cadherin knockdown clones in SUM149 cells. The use of 2 shRNA molecules minimized the effect of off-target phenotypes often observed using shRNA approaches. Efficient knockdown of E-Cadherin was observed in 2 independent SUM149 clones for each one of the E-Cadherin shRNA plasmids (shECad-65 and shECad-66) (Fig. 2b). Increased expression of mesenchymal markers, N-Cadherin, ZEB1 and vimentin, was detected in all SUM149-shECad clones compared to the control SUM149-shNT (non-target) clones (Fig. 2b). Reduction of membrane localized E-Cadherin protein was also confirmed by immunofluorescence staining (Sup. Fig. S3c). Similar reductions in β-catenin membrane localization was also observed in SUM149-shECad clones (Sup. Fig. S3c). Concomitant with upregulation of mesenchymal markers, the morphology of the SUM149-shECad clones cultured under adherent conditions was altered from a cuboidal shape in SUM149-shNT clones to a more elongated shape for SUM149-shECad clones (Fig. 2d). Although knockdown of E-Cadherin had no statistically significant effect on cell proliferation (Sup. Fig. S3a), a slight increased in Matrigel invasion was observed in SUM149-shECad clones *in vitro* (Fig. 2f).

### Over-expression of *ZEB1* in SUM149 leads induction of EMT markers

The importance of the ZEB1 transcription factor, a known repressor of E-Cadherin, in promoting EMT and enrichment of cells with a cancer stem cell phenotype has been highlighted in recent publications [19, 20]. Our recent studies identified the loss of *ZEB1* as a characteristic signature in IBC patients and pre-clinical models of IBC [21, 22]. To assess the effects of the presence of ZEB1 in IBC tumor cells, *ZEB1* over-expressing clones of SUM149 cells were generated. Forced expression of *ZEB1* by SUM149 (SUM149-ZEB1) clones lead to increased expression of nuclear ZEB1 protein and induced expression of the mesenchymal proteins N-Cadherin and vimentin (Fig. 2c). With the exception of the reduction in E-Cadherin protein (Fig. 2c), the SUM149-*ZEB1* clones phenocopied that of the SUM149-shECad clones. Similarly, there were morphological changes in SUM149-ZEB1 cells, with a switch from a cuboidal shape in control SUM149-LUC clones characteristic of epithelial phenotype to a more elongated shape for SUM149-ZEB1 clones. The presence of ZEB1 had no statistically significant effect on either cell proliferation (Sup. Fig. S3b) or on *in vitro* Matrigel invasion of SUM149-ZEB1 cells (Fig. 2g)

Taken together, these data demonstrate that either the loss of E-Cadherin or the gain of *ZEB1* induced alterations in SUM149 IBC tumor cells, suggesting that these genes directly regulate morphological alterations that have been associated with EMT. Because of the observed epithelial nature of the control clones (SUM149-shNT and SUM149-LUC clones), we will refer to them as epithelial E-SUM149 clones and similarly we will refer to SUM149-shECad and SUM149-ZEB1 clones as mesenchymal M-SUM149 clones.

### Gene profiling analysis of E-SUM149 and M-SUM149 clones

**Figure 3:**
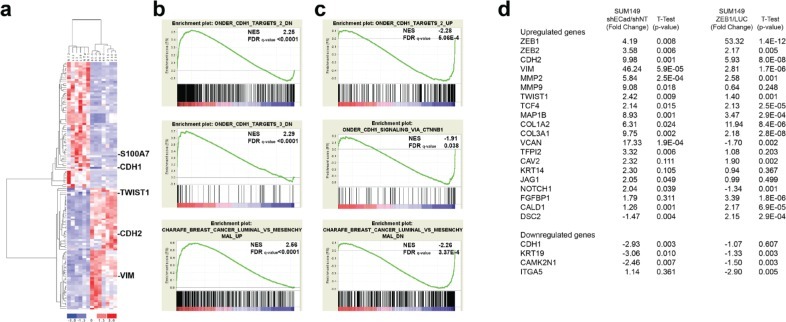
Gene profiling of E-SUM149 and M-SUM149 clones (a) Unsupervised clustering of E-SUM149 clones (SUM149-NT1, SUM149-NT2, SUM149-LUC-L3, SUM149-LUC-L39, SUM149-LUC-L42), including the parental SUM149, and M-SUM149 clones (SUM149-ShECad-65-1, SUM149-ShECad-65-2, SUM149-ShECad-66-1, SUM149-ShECad-66-2, SUM149-ZEB1-c16, SUM149-ZEB1-c30 and SUM149-ZEB1-c38). The E-SUM149 and M-SUM149 clones segregated into their respective group. Selected EMT related genes are shown and were found to be upregulated in the M-SUM149 groups. (b) GSEA analysis for genesets enriched in the E-SUM149 group, and includes genes whose expression is correlated with E-Cadherin expression (ONDER CDH1 TARGETS 2 DN, ONDER CDH1 TARGETS 3 DN and CHARAFE BREAST CANCER LUMINAL VS MESENCHYMAL UP). (c) GSEA analysis for genesets enriched the M- SUM149 clones whose expression were inversely correlated with E-Cadherin expression (ONDER CDH1 TARGETS 2 UP, ONDER CDH1 SIGNALING CTNNB1, CHARAFE BREAST CANCER LUMINAL VS MESENCHYMAL DN). (d) PCR array validation of EMT related genes. Loss of expression of E-Cadherin or overexpression of ZEB1 elicit an increased in EMT associated genes in SUM149 cells.

To further define genes that are altered in E-SUM149 and M-SUM149 cells, these clones were compared to their respective control clones by Affymetrix array analysis. Unsupervised hierarchical clustering analysis revealed, a segregation of the E-SUM149 clones (SUM149-NT1, SUM149-NT1, SUM149-LUC-L3, SUM149-LUC-L39, SUM149-LUC-L42 and including the parental SUM149) and the M-SUM149 clones (SUM149-shECad-65-1, SUM149-shECad-65-2, SUM149-shECad-66-1, SUM149-shECad-66-2, SUM149-ZEB1-c16, SUM149-ZEB1-c30 and SUM149-ZEB1-c38) (Fig. 3a). The SUM149-shECad and SUM149-ZEB1 cells phenocopied each other in terms of their overall gene expression level, similar to the results of biochemical and cellular analysis described above. Also, the control clones selected segregated with parental SUM149, indicating that the selected control clones are appropriate. We observed 391 genes that were differentially expressed, with greater than a 2-fold change (FDR<0.397%; Sup. Table 1) between E-SUM149 and M-SUM149 clones. The most differentially expressed gene in E-SUM149 clones, S100A7, also known as psoriasin (40-fold) which has previously been implicated in breast tumorigenesis [23, 24]. Using the gene set enrichment analysis (GSEA) software, numerous statistically significant redundant genesets were enriched in E-SUM149 clones and M-SUM149 clones (Table 1). Numerous genesets related to E-Cadherin status or epithelial features were enriched in control E-SUM149 clones while genesets related to mesenchymal features were enriched in the M-SUM149 clones *in vitro* (Table 1). Of note, the highest enrichment score genesets came from a group of genesets defined by Onder *et al* [25] resulting from the knockdown of E-Cadherin in the immortalized human breast epithelial cell line, HTMLR. Thus, E-SUM149 clones were enriched in genes belonging to genesets that correlate with the presence of E-Cadherin (Fig. 3b) such as Onder CDH1 Targets 1 DN and Onder CDH1 Targets 2 DN. Furthermore, a geneset associated with the luminal phenotype (Charafe Breast Cancer Luminal vs Mesenchymal Up) was also enriched in E-SUM149 clones. Conversely, genesets associated with the loss of E-Cadherin or mesenchymal phenotype were enriched in M-SUM149 clones (Table 1, Fig. 3c). The upregulation of expression of multiple EMT-associated genes by M-SUM149 clones such as *ZEB1*, *ZEB2*, *Twist1*, N-Cadherin, MMP2, MMP9 and vimentin was confirmed by quantitative PCR analysis (Fig. 3d).

**Table 1:** Genesets enriched in E-SUM149 (positive NES score) and M-SUM149 (negative NES score) clones *in vitro*

GeneSet	Normalized Enrichment Score (NES)	FDR q-value
E-Cadherin/Epithelial Genesets		
CHARAFE_BREAST_CANCER_LUMINAL_VS_MESENCHYMAL_UP	2.56	<0.0001
ONDER_CDH1_TARGETS_3_DN	2.29	<0.0001
ONDER_CDH1_TARGETS_2_DN	2.25	<0.0001
DOANE_BREAST_CANCER_ESR1_DN	2.08	0.0044
ONDER_CDH1_TARGETS_1_DN	1.82	0.050
CHARAFE_BREAST_CANCER_LUMINAL_VS_BASAL_UP	1.73	0.105
		
E-Cadherin/Epithelial Genesets		
ONDER_CDH1_TARGETS_2_UP	−2.28	5.055E-4
CHARAFE_BREAST_CANCER_LUMINAL_VS_MESENCHYMAL_DN	−2.26	3.370E-4
GU_PDEF_TARGETS_UP	−2.25	5.136E-4
SCHUETZ_BREAST_CANCER_DUCTAL_INVASIVE_UP	−2.09	0.007
CHARAFE_BREAST_CANCER_BASAL_VS_MESENCHYMAL_DN	−2.03	0.013
ONDER_CDH1_SIGNALING_VIA_CTNNB1	−1.91	0.037

### *In vivo* growth of E-SUM149 and M-SUM149 cells

**Figure 4:**
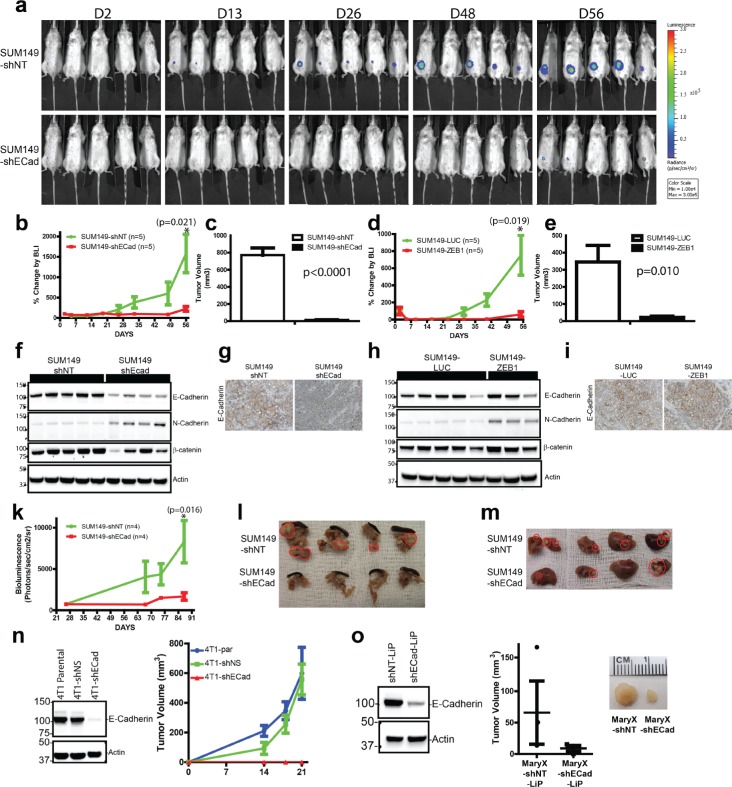
E-Cadherin is required for *in vivo* growth of SUM149, Mary-X and 4T1 breast cancer cell lines (a) Longitudinal bioluminescent imaging (BLI) of SUM149-shNT and SUM149-shECad cells growth in MFP. Similar results were obtained with SUM149-LUC and SUM149-ZEB1 clones. (b,d) M-SUM149 clones display reduced *in vivo* growth capacity. Five hundred thousand of E-SUM149 or M-SUM149 clones were implanted into MFP and BLI was measured to monitor tumor growth. BLI was performed on a weekly basis for 8 weeks. Five mice per group was used. Similar results were obtained when 50,000 cells were used (data not shown). (c,e) Reduced tumor volume of M- SUM149 clones when compared to E-SUM149 clones 8 weeks post implantation (n=5). (f,h) Western blot validation of xenograft tissue cell extract from E-SUM149 and M-SUM149 clones. SUM149-shECad tumors maintained their reduced E-Cadherin expression while retaining N-Cadherin expression. Additionally, βcatenin expression levels were correlated with E-Cadherin expression. (g) Based on immunohistochemical staining, reduced E-Cadherin staining was maintained in SUM149-shECad. (i) Immunohistochemical staining of E-Cadherin protein on SUM149-LUC and SUM149-ZEB1 tumor tissues. E-Cadherin expression was retained in the SUM149-ZEB1 tumor tissue similar to the result observed by western blot. (k) BLI of tumor burden in intra-cardiac injection metastatic model. E-Cadherin expression in SUM149 cells is required for metastatic colonization. Longitudinal study of metastatic burden of SUM149-shNT (n=4) and SUM149-shECad (n=4). Decrease metastatic foci in pancreas (l) and liver (m) of SUM149-shECad injected mice. Tumors localization within the tissues are traced in red. (n, left) Western blot showing efficient knockdown of E-Cadherin in 4T1-shECad cells. (n, right) Dramatic reduction of MFP *in vivo* growth of 4T1-shECad cells compared to either 4T1 parental or control 4T1-shNS. (o, left) Efficient knockdown of E-Cadherin using the modified pLKO-LiP vector backbone. (o, right) Reduced *in vivo* growth of Mary-X-shEcad-LiP cells compared to Mary-X-shNT-LiP

The next studies evaluated the ability of E-SUM149 and M-SUM149 clones to form tumors *in vivo* when injected into the mammary fat pad (MFP) of NOD.Cg-Prkdcscid Il2rgtm1Wjl/SzJ mice. Five hundred thousand cells were implanted into the MFP and growth was monitored by bioluminescent imaging (BLI) on a weekly basis for 8 weeks (Fig. 4a). The E-SUM149 clones displayed exponential growth, compared to the lack of *in vivo* growth by M-SUM149 clones (Fig. 4a,b,d). Similar results were observed when 50,000 cells were implanted (data not shown). The lack of growth of M-SUM149 tumor cells, as detected by BLI, was consistent with the reduced tumor volumes observed at the time of necropsy for M-SUM149 tumors clones compared to the volume of tumors formed by epithelial E-SUM149 control clones (Fig. 4c,e). Western blot analysis performed on xenograft tumor extracts confirmed the maintenance of decreased E-Cadherin expression in the SUM149-shECad xenograft tissue, with the concomitant upregulation of N-Cadherin (Fig. 4f). This pattern is similar to the one observed in 2D *in vitro* culture of SUM149-shECad cells (Fig. 2b). SUM149-ZEB1 xenograft tissues also display a similar pattern of gain of N-Cadherin protein as was observed in 2D *in vitro* culture (Fig. 4h). Immunohistochemical staining was performed using xenograft tissues to confirm the status of E-Cadherin expression (Fig. 4g.i). E-Cadherin was expressed in sections of tissue from control E-SUM149 tumors and was reduced in SUM149-shECad but not in SUM149-ZEB1 sections, similar to what is observed in Western blot studies using *in vitro* cultured cells. We also assessed the role of E-Cadherin on the *in vivo* metastatic potential of SUM149-shNT and SUM149-shECad clones by intracardiac injection of tumor cells as a model of hematogenous dissemination of cancer cells. There was reduced whole body metastatic burden by BLI measurements when SUM149-shECad cells were injected as compared to SUM149-shNT cells (Fig. 4k). Specifically, there was reduced metastatic foci and size in metastasis to the pancreas (Fig. 4l) and liver (Fig. 4m). Thus, collectively these results demonstrate that E-Cadherin is required for both primary and metastatic tumor growth in SUM149 cells.

### Lack of *in vivo* growth in 4T1 and Mary-X cells with reduced E-Cadherin expression

To evaluate the generality of the observation that loss of E-Cadherin regulated reduced *in vivo* growth, further *in vivo* experiment were performed using the mammary mouse cell line 4T1 and the triple negative IBC cell line Mary-X. Efficient E-Cadherin knockdown was obtained in both cell lines (Fig. 4n,o) and reduced *in vivo* growth was observed in both 4T1-shECad and Mary-X-shECad cell (Fig. 4n,o). These results indicate a similar requirement for E-Cadherin for *in vivo* growth of 4T1 and Mary-X cells, which is consistent with the results observed in the SUM149 cells.

### Microarrays and GSEA of xenograft tissues

To identify differentially expressed genes between E-SUM149 and M-SUM149 tumor tissues, gene profiling studies were performed. RNA isolated from tumor tissues and analyzed with the Affymetrix U133 Plus 2 chip and GSEA was performed. There were greater than 500 genes that were differentially expressed between the E-SUM149 and M-SUM149 clones *in vivo* (>2=fold, FDR=0.0%; Sup. Table 2). In the control E-SUM149 clone xenograft tissues, the most prevalent geneset identified were involved in the response to hypoxia (Table 2). Finally, genesets involving E-Cadherin signaling were also enriched in the control E- SUM149 clone tumors, similar to what was observed *in vitro*. Conversely, genesets enriched in the M-SUM149 clone groups consisted of gene related to proliferation and cell cycle (Table 2). Genesets involving DNA replication were also enriched in this group.

**Table 2:** Genesets enriched in E-SUM149 (positive NES score) and M-SUM149 (negative NES score) clones in vivo

GeneSet	Normalized Enrichment Score(NES)	FDR q-value
Hypoxia Genesets		
HARRIS_HYPOXIA	2.63	<0.0001
WINTER_HYPOXIA_METAGENE	2.40	<0.0001
WEINMANN_ADAPTATION_TO_HYPOXIA_DN	2.35	<0.0001
LEONARD_HYPOXIA	2.34	<0.0001
ELVIDGE_HYPOXIA_BY_DMOG_UP	2.33	<0.0001
WINTER_HYPOXIA_UP	2.24	<0.0001
E-Cadherin Genesets		
ONDER_CDH1_TARGETS_2_DN	2.45	<0.0001
ONDER_CDH1_TARGETS_3_DN	2.251	<0.0001
Proliferation Genesets		
ROSTY_CERVICAL_CANCER_PROLIFERATION_CLUSTER	−2.92	<0.0001
SOTIRIOU_BREAST_CANCER_GRADE_1_VS_3_UP	−2.89	<0.0001
KANG_DOXORUBICIN_RESISTANCE_UP	−2.68	<0.0001
GRAHAM_NORMAL_QUIESCENT_VS_NORMAL_DIVIDING_DN	−2.50	<0.0001
MOLENAAR_TARGETS_OF_CCND1_AND_CDK4_DN	−2.47	<0.0001
ODONNELL_TFRC_TARGETS_DN	−2.47	<0.0001
BLUM_RESPONSE_TO_SALIRASIB_DN	−2.36	<0.0001
REACTOME_G2_M_CHECKPOINTS	−2.26	<0.0001
DNA Replication Genesets		
REACTOME_DNA_STRAND_ELONGATION	−2.23	<0.0001
REACTOME_MITOTIC_M_M_G1_PHASES	−2.21	1.88E-5
REACTOME_MITOTIC_PROMETAPHASE	−2.21	1.84E-5
REACTOME_ACTIVATION_OF_ATR_IN_RESPONSE_TO_REPLICATION_STRESS	−2.16	2.99E-5
KAUFFMANN_DNA_REPAIR_GENES	−2.14	9.73E-5
KEGG_DNA_REPLICATION	−2.08	3.29E-4
REACTOME_ACTIVATION_OF_THE_PRE_REPLICATIVE_COMPLEX	−2.07	3.43E-4
REACTOME_CELL_CYCLE_CHECKPOINTS	−2.07	3.78E-4

### Validation of potential target by western blot and immunohistochemistry staining

**Figure 5:**
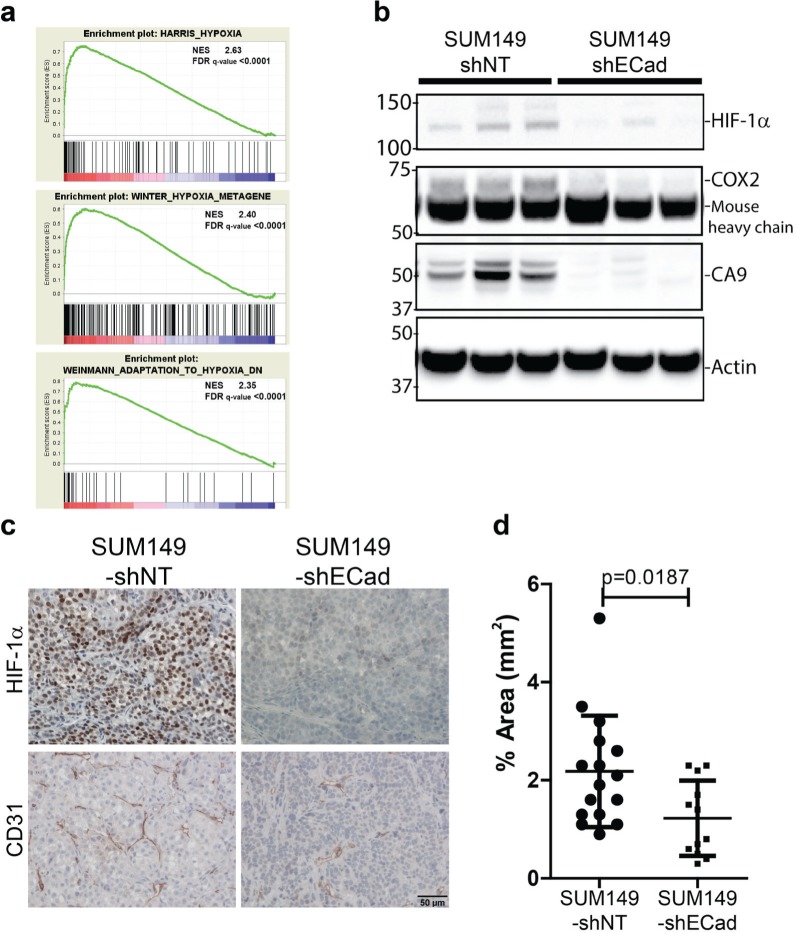
Lack of expression of genes involved in the hypoxic response in M-SUM149 clones tissues (a) GSEA demonstrated diminished expression of genes involved in hypoxic responses in M-SUM149 xenograft tissues. Three hypoxia-related signature with the associated enrichment score (ES) and the associated false discovery rate (FDR). (b) Western blot validation of hypoxia related proteins on SUM149-shNT and SUM149-shECad tissues. Decrease expression of HIF-1α, COX2, CA9 proteins was observed in SUM149-shECad tissues. (c) Immunohistochemical detection of HIF-1α protein and CD31-based microvessels in SUM149-shNT and SUM149-shECad tumor tissues. (bar=50uM). (d) Quantification of CD31 expression in SUM149-shNT and SUM149-shECad tumor tissues.

The GSEA analysis suggested that the response to hypoxia is important for *in vivo* growth of E-SUM149 clones (Fig. 5a). To validate these GSEA results, we performed western blot and IHC of HIF-1α on xenograft tissues (Fig. 5b). Based on western blot analysis, HIF-1α protein was reduced in the M-SUM149 tissues (Fig. 5b), as were the hypoxia response proteins, Carbonic Anhydrase 9 (CA9), and prostaglandin-endoperoxide synthase 2 (prostaglandin G/H synthase and cyclooxygenase) (PTGS2/COX2). By IHC, HIF-1α protein was detected in tissue sections from E-SUM149 clones while M-SUM149 clones had a dramatic reduction in HIF-1α staining (Fig. 5c). Furthermore, diminished HIF-1α protein was associated with reduced endothelial CD31 staining and microvessel density in M-SUM149 tissues (Fig. 5c,d). These results suggest that M-SUM149 clones display altered responsiveness to stress and/or hypoxia *in vivo*, associated with decreased vascularization, which is consistent with the recognized role of HIF-1α in regulation of tumor angiogenesis [26, 27]

### Rescue of *in vivo* growth defect of SUM149-shECad clones with HIF-1α

**Figure 6:**
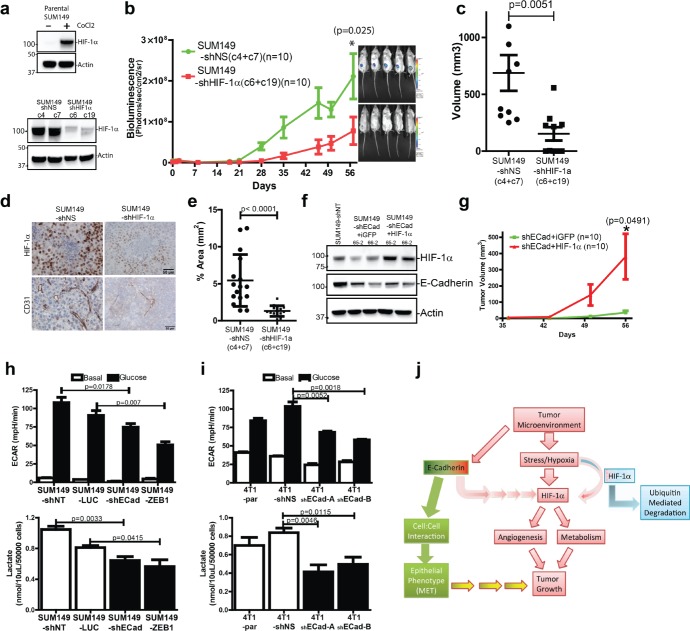
HIF-1α functions are central to the growth of E-Cadherin expressing breast cancer cells *in vivo* (a) (*top*) Knockdown of HIF-1α in SUM149. HIF-1α is only detectable in the presence of CoCl_2_, in SUM149 parental cells cultured *in vitro*. (*bot*) Western blot of HIF-1α on selected control clones ((SUM149-shNS clone 4 (c4) and -clone 7(c7)) and HIF-1 a knockdown clones (SUM149-shHIF-1 α-clone 6 (c6) and -clone 19 (c19)) in the presence of 500uM CoCl_2_ for 3 hours. (b) Reduce tumor growth in SUM149-shHIF-1 a when implanted into the MFP of mice. The average BLI of the control cell clones (SUM149-shNS-c4 (n=5), SUM149-shNS-c7 (n=5)), HIF-1 α knockdown clones (SUM149-shHIF-1 α-c6 (n=5) and SUM149-shHIF-1 α-c19 (n=5)) are shown. Representative BLI images on day 56 prior to sacrifice (c) Tumor volumes of SUM149-shNS and SUM149-shHIF-1α xenografts at sacrifice (p=0.0051). (d) Immunohistochemical staining of HIF-1α adjacent to hypoxic area. Both the presence and amount of HIF-1α protein surrounding hypoxic areas is greatly reduced in the SUM149-shHIF-1α tissues. The CD31 stained microvessels were also reduced in the SUM149-shHIF-1α xenograft tissues. (e) Quantification of CD31 expression in SUM149-shNT and SUM149-shECad tissues. (f) Generation of HIF-1α overexpressing SUM149-shECad. Western blot levels of HIF-1α in CoCl_2_ treated SUM149-shECad clones (65-2 and 66-2). (g) Overexpression of HIF-1α in SUM149-shECad rescue its growth defect in the MFP of mice (n=8-9 mice per group). (h) (top) Reduce extracellular acidification rate (ECAR) as measured by the Seahorse instrument following the injection of 10mM glucose in M-SUM149 clones. ECAR values were used as surrogate of glycolysis and were validated by direct measurements of extracellular measurements of L-lactate (bot). p-values are from an unpaired two-tailed T-test (j) ECAR (top) and extracellular L-lactate measurements (bot) of 4T1-parental, 4T1-shNS and 4T1-shECad cells. (j) Schematic model of E-Cadherin-mediated signaling *in vivo*. In the presence of E-Cadherin. SUM149 cells induces HIF-1α protein expression which provides a survival advantage to tumor cells, allowing them a means to adapt to a hypoxic microenvironment and supports their survival and growth.

To validate the importance of HIF-1α in SUM149 growth *in vivo*, HIF-1α was knocked down using shRNA in the SUM149-LUC39 clone that was used previously (Fig. 3d). Two non-silencing (shNS) and 2 shHIF-1α single clones were isolated and evaluated for HIF1α protein by western blotting. Under non-stressed *in vitro* culture conditions, HIF-1α was only detectable when treated with CoCl_2_, which prevents HIF-1α protein degradation (Fig. 6a (top)). Following a 500uM CoCl_2_ challenge for 3 hrs, reduced HIF-1α protein stabilization was observed in SUM149-shHIF-1α clones (Fig. 6a (bot)). SUM149-shNS (clones 4 and 7) and SUM149-shHIF-1α (clones 6 and 9) cells were injected into the mammary fat pad of mice and their growth was monitored by BLI. Reduced expression of HIF-1α in SUM149 cells (SUM149-shHIF1α) led to reduced growth *in vivo* compared to the control SUM149-shNS cells (Fig. 6b). Tumor volumes at 8 weeks post-implantation were consistently reduced in the SUM149-shHIF1α groups (Fig. 6c). Immunhistochemical staining for HIF-1α and CD31 proteins confirmed that HIF-1α protein levels were reduced. (Fig. 6d), which was also associated with a reduction in the number of CD31-positive endothelial cells and microvessel density in tissue sections from the SUM149-shHIF1α xenografts (Fig. 6d,e). To confirm the role of HIF-1α in mediating the growth defect in E-Cadherin knockdown SUM149 cells. HIF-1α was over-expressed in SUM149-shECad clones and injected into the MFP of mice (Fig. 6f,g). Over-expression of HIF-1α in SUM149-shECad clones was sufficient to rescue the growth defect of SUM149-shECad clones. Taken together, these results support a model of E-Cadherin mediated signaling *in vivo* in which E-Cadherin is required for the regulation of angiogenesis through HIF-1α induction.

### Reduced glycolysis in E-Cadherin knockdown cells

We next evaluated the effects of the lack of E-Cadherin expression on metabolic activity in SUM149 and 4T1 cells. Using the Seahorse Flux Analyzer instrument, we measured the extracellular acidification rate (ECAR) as a surrogate measurement of glycolysis. We observed reduced glycolysis in both M-SUM149 and 4T1-shEad clones when challenged with glucose (Fig. 6h,i). The reduced ECAR observed in the M-SUM149 and 4T1-shEad clones was validated with extracellular measurements of L-lactate, a byproduct of glycolysis. A reduction in L-lactate production was observed in both the M-SUM149 and 4T1-shEad clones.

## DISCUSSION

Although the induction of EMT and loss of E-Cadherin is believed to be an essential primary step in the initiation of metastasis, it is well accepted that tumors are quite heterogeneous and loss of E-Cadherin is not always associated with increased invasive behavior [15]. Similarly, maintenance of E-Cadherin is not detrimental to invasion and metastasis (see below). The plasticity of the linked reversible processes of EMT and MET suggests that cancer cells retain a control in the degree of cellular re-programming and may select EMT features such as invasion while maintaining epithelial features such as E-Cadherin expression. While recent studies have focused on the loss of E-Cadherin as part of the process of EMT, other studies suggest that the maintenance of E-Cadherin is likely to function as an oncogene depending of the cellular context, similar to reports of the dichotomous tumor-suppressing or tumor-promoting function of Transforming growth factor beta (TGFβ) [28].

As an example of a tumor type that is well recognized to retain E-Cadherin as part of its molecular signature, IBC is the most aggressive form of breast cancer and is characterized by an earlier age of onset (<52 years) than other variants of breast cancer and by the high rate of metastasis [14]. Molecular analysis of IBC specimens have demonstrated that the molecular sub-types are similar to the ones described for non-IBC breast cancer, with a preponderance of IBC patients with either triple negative or Her2 tumors [29]. Regardless of molecular subtype, E-Cadherin expression has been documented to be one of the few molecular markers that are consistently present in IBC patient biopsy samples [30, 31]. Similarly, we saw high expression of E-Cadherin in 6 out of the 7 IBC cell lines examined, which represent all currently available pre-clinical models of IBC. Large scale breast cancer association of high E-Cadherin expression in basal breast tumors suggests a poor RFS prognosis, while no significant clinical association was observed for the EMT marker N-Cadherin. Furthermore, the poor RFS prognosis was also observed when unselected breast tumors were interrogated (Sup Fig. S1). A similar poor prognosis value of E-Cadherin expression in breast and prostate cancer were recently described [8, 32].

In the present study, a dramatic effect on *in vivo* growth was observed in 3 distinct cell systems, SUM149, Mary-X and 4T1 with E-Cadherin knockdown. Similarly, over-expression of ZEB1 in SUM149 elicit a similar *in vivo* growth reduction. Detailed biochemical and molecular analysis of the SUM149-shECad and SUM149-ZEB1 clones suggested loss of E-Cadherin was associated with induction of an EMT program which included *in vitro* morphological changes, expression of the mesenchymal proteins N-Cadherin, vimentin and expression of the nuclear transcription factor ZEB1, a known repressor of E-Cadherin. Furthermore GSEA analysis of the E- and M-SUM149 clones identified as the top rank up and down genesets, as reported by Onder et al [25], in which E-Cadherin was reduced in the H-RAS immortalized human breast epithelial cells (HMLER), indicating the conservation in the transcriptional signaling of E-Cadherin between HMLER and E-SUM149 clones. The similarity in transcriptional profiling between HMLER and E-SUM149 clones did not extend to their *in vivo* growth capacity, where the HMLER-shECad cells grew at similar rate as the control HMLER cells. The lack of *in vivo* growth of the M-SUM149 clones despite the induced EMT is at odds with the EMT/cancer stem cell (CSC) hypothesis where the EMT gene signature is enriched in cancer stem cells that express CD44^high^/CD24^low^ (CSC) [33]. While both SUM149 and Mary-X cells express E-Cadherin, they are also enriched for CSCs [34]. Finally, E-Cadherin was also required for the establishment of metastatic foci of SUM149 cells in the hematogenous dissemination mouse model.

The appreciation of the importance of the reversion to MET for growth and survival in highly metastatic cell lines has recently been described using models of prostate and bladder cancers [6-8]. Interestingly, overt EMT in the PC3 prostate cancer and the TSU-Pr1 bladder cell lines was demonstrated to lead to reduced tumor-initiating or CSC potential compared to their epithelial sublines [6, 35], which is consistent with our *in vivo* results of reduced tumor-initiating potential in the M-SUM149 clones, Mary-X-shEcad and 4T1-shECad cells.

Thus the requirement for E-Cadherin or MET for tumor growth appears to be subtype or context dependent, and is likely modulated by genetic factors. In the case of aggressive tumors such as ovarian or breast cancer, E-Cadherin expression appears to confer a survival advantage to tumor cells in both the primary and metastatic settings. In the case of mesenchymal-type of cancer, overt EMT appears to represent a primary mechanism for acquisition of an invasive phenotype associated with enrichment for CSCs. Conversely, some epithelial-type of cancer rely on maintenance of an epithelial phenotype or reversion to MET for their tumorigenic activity, metastatic capability and maintenance of populations of CSCs. Overall, this reinforces the well accepted concept of tumor heterogeneity and cellular plasticity where multiple pathways can lead to similar outcomes

To identify the mechanism of the reduced *in vivo* growth in E-Cadherin knockdown cells, microarray gene profiling and GSEA from isolated E-and M-SUM149 clone xenografts was performed. The top genesets in E-SUM149 clones were hypoxia and E-Cadherin/CDH1 regulated genesets. We validated the lack of hypoxia response by of M-SUM149 xenografts by reduced expression of HIF-1α, CA9, and COX2 by western blot analysis and reduced CD31 endothelial density by immunohistochemistry. Furthermore, the change in hypoxia-related genes was only observed in the *in vivo* SUM149 xenograft setting and was not present when *in vitro* cultured SUM149 cells were examined, demonstrating the importance of the microenvironment to the interactions between E-Cadherin signaling and hypoxia responsive genes (Fig. 6j). The role of HIF-1α in SUM149 MFP growth was confirmed by reduced growth of HIF-1α knockdown in SUM149 clones compared to the control clones *in vivo*, which was accompanied by reduced in the number of CD31 expressing endothelial cells, highlighting the crucial function of HIF-1α in regulating tumor vascularization and angiogenesis [29]. Furthermore, re-expression of HIF-1α in SUM149-shECad clones rescued the tumor growth defect *in vivo*.

The ability to adapt to the microenvironment is likely an initial survival mechanism that cancer cells must activate in order to survive both the harsh conditions that exist within the primary tumor and in metastatic microenvironments. Perhaps not surprisingly, recent studies described the enrichment of hypoxia signaling in both IBC as well as in samples from patients with triple negative breast cancer [36, 37]. Hypoxia is one of the most universal hallmarks of cancer and the transcription factor HIF-1α mediates a majority of cancer-associated transcriptional changes [38-40]. Given the importance of HIF-1α in cellular metabolism and particularly aerobic glycolysis that is prevalent in tumors as part of the defined Warburg effect [41, 42], we observed a decreased in glycolysis as measured by ECAR in M-SUM149 and 4T1-shEcad clones associated with independently measured reductions in secreted L-lactate, a byproduct of glycolysis. This set of results points to the importance of cellular metabolism in promoting tumor growth, with the reduce glycolysis in M-SUM149 and 4T1-shECad clones likely an important contributor to their poor *in vivo* growth.

The present results implicating HIF-1α and hypoxia signaling in mediating growth, metastasis and survival of breast tumor cells is consistent with HIF-1α as an important therapeutic target however thus far its exploitation has yet to be realized [38-40]. Our discovery of the E-Cadherin/HIF-1α/glycolysis axis in tumors opens the possibility of novel therapeutic strategies that would potentially target both primary tumors as well as metastatic lesions. The link between E-Cadherin and HIF-1α/glycolysis is novel and suggests that E-Cadherin is a bona-fide signaling molecule that indirectly regulates HIF-1α which ultimately mediates not only metabolic responses of tumor cells (ie, glycolysis) but also regulates *in vivo* growth in part due to impacting responses in the microenvironment such as interactions with vascular endothelium during tumor vascularization and tumor angiogenesis. While the exact molecular mechanisms at play remain to be identified, the signaling activities of E-Cadherin and the downstream effectors involved in the responses to hypoxia are clearly dependent on the *in vivo* microenvironment. Because of the dramatically reduced growth of the M-SUM149, Mary-X-shEcad and 4T1-shECad clones observed *in vivo* in the present studies, the development of novel therapeutics against E-Cadherin and HIF-1α/glycolysis represent an attractive approach for the treatment of IBC and possibly in cancers that retain an epithelial phenotype typified by E-Cadherin expression in general. Our recent results demonstrating that HDAC inhibitors target both E-Cadherin and HIF-1α in breast tumors point to the promise of this type of therapeutic approach [43].

## MATERIALS AND METHODS

### *In vivo* Studies

Cells were injected at a concentration of five hundred thousand cells into the 4th mammary fat pad of NOD.Cg-Prkdcscid Il2rgtm1Wjl/SzJ mice (n=5-10 per clone; Jackson Labs) and their growth monitored by weekly luciferase bioluminescent imaging or caliper measurements. Animals were sacrifice at 8 weeks post implantation and tumors were either fixed with 10% neutral buffered formalin or stored in Trizol (Invitrogen) or in T-Per lysis buffer(Thermo Scientific). Tumor volumes were calculated using the following formula, Volume = (width)^2^ × length/2

For metastasis studies, 200,000 cells were injected into the left ventricle of NOD.Cg-Prkdcscid Il2rgtm1Wjl/SzJ mice. BLI was performed following the injection and on a weekly basis for 6-8 weeks to monitor tumor burden as described in [44]

### Cell Lines

MDA-MB-231, MCF-7 and 4T1 cell lines were purchased from ATCC. The SUM159, SUM149 and SUM190 cell lines were purchased from Asterand. The MDA-IBC-3 cell line was generated from the pleural effusion of an IBC patient and developed by Dr. W. Woodward at The University of Texas MD Anderson Cancer Center [45]. The FC-IBC01 and FC-IBC02 cell lines was generated from the pleural effusion of IBC patients and maintained as suspension cultures in MEBM medium (Lonza) supplemented with B27 supplements (Invitrogen), 20 ng/ml recombinant EGF (Invitrogen), 20ng/ml basic FGF (Invitrogen), 4 ug/ml heparin (Sigma), L-Glutamine (Invitrogen) and the antibiotics penicillin and streptomycin (Invitrogen). The SUM149, SUM190 and MDA-IBC-3 cells were maintained in Ham F12 Nutrient Mixture (Invitrogen) supplemented with 10% fetal bovine serum (FBS; Invitrogen), insulin (1 mg/ml; Sigma-Aldrich), and hydrocortisone (1 mg/ml; Sigma-Aldrich). MCF-7 and MDA-MB-231 cells were cultured in Dulbecco's modified Eagles and F12 medium (DMEM/F12; Invitrogen) supplemented with 10% FBS. SUM159 cells were grown in DMEM supplemented with 10% FBS.

### Cell Proliferation

Cell Proliferation was assayed using the ProMega CellTiter 96® AQueous One Solution Cell Proliferation Assay according the manufacturer's instructions. Briefly, cells were seeded into a 96 well plate at 1500 cells. Experiments were terminated 24, 48 and 72 hours post-plating and processed according to the manufacturer's instructions and read at 490 nm on a BioTek plate reader. Data analysis was performed using GraphPad 5.0 (GraphPad Software).

### Invasion

The FluoroBlok™ cell culture inserts (8.0uM; BD Falcon) were coated with 300 μl of Matrigel™ (1:1 Dilution) and allowed to set for 2hr at 37°C. Coated inserts were placed in the 24 well companion plate containing 1mL of complete DMEM/F12 medium. In the top half of the insert 50,000 cells were plated in 0.25mL of complete DMEM/F12 medium. Plates were then incubated at 37°C for 24hr to allow invasion to occur. Invaded cells were stained with 1 uM Calcein-AM (Invitrogen) for 30 minutes at 37°C. Fluorescence signal of invaded cells is read at wavelengths of 494/517 nm (Ex/Em) on a bottom-reading fluorescent BioTek plate reader.

### Generation of knockdown and overexpressing cells

E-Cadherin knockdown lentiviral shRNA constructs (TRCN0000039665 and TRCN0000039666; thereafter referred to as shECad-65 and shECad-66 respectively) were purchased from Sigma. The non-targeting shRNA vector (SHC002) was used as a negative control. Lentivirus particles were generated in 293FT cells (Invitrogen) by co-transfecting the shRNA construct (3ug) and the ViraPower DNA mix (9ug) with Fugene6 (Roche). Lentiviral particles were collected 48 hours post transfection, filtered through a 0.45uM filter (Millipore). SUM149, Mary-X and 4T1 cells were transduced in the presence of 8 ug/ml Polybrene (Sigma) for 48 hours. Transduced cells were selected by puromycin (Invitrogen) selection (1 ug/ml). Single clones were selected for transduced SUM149 and 4T1 cells by serial dilution and 2-3 single clones for each shRNA construct further analyzed. The pGIPZ-shHIF-1α constructs were purchased from the shRNA and ORFeome Core Facility (MD Anderson Cancer Center, Houston, TX). Multiple shHIF-1α constructs were tested for efficiency of HIF-1α knockdown and 2 constructs (Clone ID: V2LHS_132150 and V3LHS_374856) were selected for viral production. Lentiviral particles were generated with the Trans-Lentiviral™ Packaging Kit (Open Biosystems, Huntsville, AL) according to the manufacturer's instruction. The SUM149-LUC (Clone 39) was transduced with the viral particles and selected 3 days later with 1ug/ml puromycin. Single clones were then selected by serial dilution and screen by western blot for HIF-1α under 500uM CoCL2-treated cells.

The cDNA for ZEB1 was purchased from Open Biosystems (MHS4426-98361372) and amplified with the following primers: ZEB1-FTAGACTGCCGGATCCATGAAAGTTACAAATTATAATACT and TAGCGGCCGCTTAGGCTTCATTTGTCTTTTCTTCA primers. The amplification was carried out using TaKaRa LA Taq DNA polymerase in order to minimize PCR mutations. The PCR product was precipitated then digested with BamHI and NotI. The digested DNA was agasose purified using Qiagen Qiaex II gel purification kit. The digested DNA was then ligated to the BamHI and NotI digested pBMN-iGFP vector (Addgene). A similar approach was performed for the generation of the Luciferase construct (pBMN-LUC-GFP) with the following primers: LUC+BamHI-F: ctagactgccggatccatggaagacgccaaaaaca; LUC+NotI-R: atttacgtagcggccgcttacacggcgatctttccgccct. The pLKO-shNT-LiP and pLKO-shECad-LiP constructs were generated by PCR. Primers with 5' BamHI were generated to amplify by PCR the Luciferase and IRES moiety from the pBMN-LUC-IRES-LUC. The PCR product was gel purified and digested with BamHI and ligated to BamHI-digested pLKO-NT (Sigma; SHC002) and pLKO-shECad (Sigma; TRC0000039666) to generate the pLKO-shNT-LiP and pLKO-shECad-LiP plasmids respectively. The integrity and orientation of the insert was confirmed by sequencing.

Generation of luciferase reporter cell lines. SUM149 clones (both E-Cadherin knockdown or ZEB1-overexpressing sets) were transduced with commercially available Luciferase (firefly)-2A-RFP (Bsd) lentiviral particles (GenTarget) and selected with 1ug/ml Blasticidin (Invitrogen, Carlbad, CA).

Overexpression of HIF-1α construct was generated by PCR. The following primers, HIF1A-Foward oligo: gatcGGATCCAGAC ATCGCGGGGA CCGATTCACC AT and HIF1A-Rev oligo: gatcGCGGCCGCTC AGTTAACTTG ATCCAAAGCT were purchased from IDT. The HIF-1α cDNA (SC119189) was purchased from Origene. The PCR was performed with Takara's LA Taq DNA Polymerase (Fisher) and digested with BamHI and NotI and subcloned into BamHI and NotI digested pBMN-iGFP (Addgene). The resulting construct was sequenced to confirm the integrity of the HIF-1α cDNA. The resulting retrovirus construct, pBMN-HIF-1α-IRES-GFP, was transfected into Pheonix cells packaging cells (Allele Biotechnology) for the generation of virus particles as described in Chu *et al* [44]. SUM149-shECad-65 and SUM149-shECad-66 cells were transduced with either viral particles encoding pBMN-HIF-1α-IRES-GFP or pBMN-IRES-GFP and transduced cells selected by FACS sorting for GFP. A supplemental table listing all the clones used in this study is available (Sup. Table 3).

### Cell Extract Preparation

Cell extracts were prepared from sub-confluent cells grown in 10cm plates. Briefly, the cells were washed once with PBS and 200uL of M-PER Mammalian Protein Extraction Reagent (Thermo Fisher Scientific) supplemented with Complete ULTRA Tablets protease inhibitors (Roche) was added to the plate. Cells were scrapped off with a plastic cell scrapper and transferred to a 1.5ml tube. The insoluble materials were cleared by centrifugation.

Tissues were resuspended in T-PER Tissue Protein Extraction Reagent (Thermo Fisher Scientific) supplemented with Complete ULTRA Tablets protease inhibitors (Roche) and phosphatase inhibitors Cocktail 2 and Cocktail 3 (Sigma-Aldrich). Tissue extracts were prepared with a mortar and pestle. The cell extract was cleared from insoluble extracts by centrifugation and sonicated (Misonix XL-2000) to shear contaminating DNA.

### Western blot analysis

Protein concentrations were performed with to the Bio-Rad DC Protein Assay Kit (Bio-Rad). 30-50 ug of proteins were loaded and separated by SDS-PAGE (4-12% NuPAGE® Bis-Tris Precast Gels (Invitrogen) followed by western blotting [44]. Antibodies against E-Cadherin, ZEB1 and vimentin (Cell Signaling), N-Cadherin and OB-Cadherin (Invitrogen), actin (Sigma), COX2 (Cayman Chemical), HIF-1α (BD Biosciences), CA9 (R&D Systems), Glut-1 (Millipore) and β-catenin (Santa Cruz Biotechnology) were all used at 1:1000 dilution. HRP-linked secondary antibodies against mouse and rabbit were purchased from Amersham. An Alpha Imager gel instrument (ProteinSimple) was used to quantify the western blot signal.

### Metabolomics studies

Extracellular acidification rates (ECAR) were measured with the XF96 extracellular flux analyzer. Wells were coated with rat tail collagen I (BD 354236) at 50ug/ml (100uL per well). Cells were plated at 40000-50000cells per wells on collagen coated wells. ECAR measurements were performed with the XF Glycolysis test kit using the manufacturer's instruction. Extracellular L-lactate concentration were measured using the Lactate assay kit (Biovision) according to the manufacturer's instruction.

### Immunohistochemistry staining

Tissues were fixed in 10% Formalin (Fisher) overnight and embedded in paraffin. Four micron sections were de-paraffinized and re-hydrated using standard protocol. Endogenous peroxidase was inactivated a 0.3% hydrogen peroxide in methanol for 5 min. Following a 5 minutes tap water wash, antigen retrieval was performed with Target Retrieval Solution (Dako) using a conventional microwave. Slides were allowed to cool to room temperature slowly and rinsed with tap water for 5 minutes. Sections were permealized with 0.2% Triton X100 in PBS for 10 minutes and rinsed 3 times with PBS. Sections were blocked with 2.5% normal horse serum and 1% IgG-free BSA (Jackson ImmunoResearch, West Grove, PA) for 30 minutes. Primary antibodies were diluted to the appropriate concentration in 0.25% Normal horse serum and 0.1% IgG-free BSA and incubate overnight at 4 C. Rabbit antibodies against E-Cadherin (24E10; 2000x) was purchased from Cell Signaling Technology. Monoclonal antibody against HIF-1α (1000x) was purchased from BD Bioscience. Rabbit anti-ZEB1 (1000x) was purchased from Bethyl. CD31 (1000x) antibody was purchased from Abcam. The sections were then washed 3 times with 0.1% Triton X100 in TBS and the appropriate ImmPRESS™ Anti-Mouse Ig (Vector Labs, Burlingame, CA), the ImmPRESS™ Anti-Rabbit Ig (Vector Labs) secondary antibody was added to the section and incubated at room temperature for 60 minutes. The slides were washed 3 times with 0.1% Triton X100 in TBS and developed with the ImmPRESS Peroxidase Polymer Detection Reagents (Vector Labs). Following a 5 minutes tap water, sections were counterstained for 2 minutes with Vector® Hematoxylin (Vector Labs, Burlingame, CA). Slides were dehydrated with sequential washes in 70%, 95% and 100% ethanol and cleared in xylene and mounted with Fisher Scientific Permount Mounting Medium (Fisher Scientific). Images were taken with a Nikon microscope. Quantification of DAB immunostaining was performed using the NIS Element software from Nikon. Briefly 10 random region located at the periphery of the tumor was captured with a 20X objective (n=3 mice for each group). A threshold setting was selected and kept constant for the analysis of all the images. The threshold was set as to detect at least 70% of the specific DAB signal.

### Microarray Preparation and Analysis

RNA was prepared using Trizol (Invitrogen) according to the manufacturer's instructions. The quality of the isolated RNA is assessed using an Agilent Bioanalyzer instrument and the quantified using a Nanodrop instrument. One ug of total RNA was reverse transcribed by using GeneChip® One-Cycle cDNA Synthesis Kit (Affymetrix, P/N 900431). Briefly, the first-strand cDNA was generated using a T7 Oligo (dT) promoter. Following RNase H-mediated second-strand cDNA synthesis, the double-stranded cDNA is purified and serves as a template in the subsequent *in vitro* transcription (IVT) reaction. The IVT reaction (GeneChip® IVT Labeling Kit Affymetrix, P/N 900449) synthesized cRNA that incorporates a biotin-conjugated nucleotide. The cRNA is then purified (using GeneChip® Sample Cleanup Module Affymetrix, P/N 900371). The hybridization cocktail (including the fragmented target (cRNA) and probe array controls (GeneChip® Hybridization Control Kit (Affymetrix, P/N 900454) is then hybridized to the probe array for 16 hours at 45C in the hybridization oven 640 (Affymetrix, P/N 800138) and rotate at 60 rpm. Immediately following hybridization, the probe array undergoes an automated washing and staining protocol on the fluidics station 400 (GeneChip® Expression Wash, Stain and Scan User Manual, P/N 702731) The processed probe is scanned using the GeneChip® Scanner 3000, The scanner is controlled by Affymetrix® GeneChip® Command Console® 3.0. The software defines the probe cells and computes intensity for each cell. Each complete probe array image is stored in a separate data file identified by the experiment name and is saved with a data image file (.dat) extension.

### Microarray analysis

Microarray data were obtained from 2-3 independent clones for each construct for the *in vitro* studies and three independent biological replicates for the *in vivo* studies. The arrays were normalized using RMAExpress standalone software (http://rmaexpress.bmbolstad.com/) using the default setting. The normalized data was analyzed with the Significance Analysis of Microarrays (SAM) software (http://www-stat.stanford.edu/~tibs/SAM/) and Gene Set Enrichment Analysis (GSEA) software (http://www.broadinstitute.org/gsea/index.jsp).

### Statistics

Unless otherwise noted, Graph Pad was used for statistical analysis. Results are expressed as mean +/− SEM. A 2-tailed Student's *t* test was applied for statistical analysis.

## Supplementary Figures and Tables




