# Supplementary material for: The Paradox of E-Cadherin: Role in response to hypoxia in the tumor microenvironment and regulation of energy metabolism
Source: Oncotarget. 2013 Mar 21;4(3):446–62. doi: 10.18632/oncotarget.872 (PMC3717307; doi:10.18632/oncotarget.872)
Supplement: Supplementary file 1 [file oncotarget-04-446-s001.pdf]

The Paradox of E-Cadherin: Role in response to hypoxia in the tumor microenvironment and regulation of energy metabolism - Chu et al

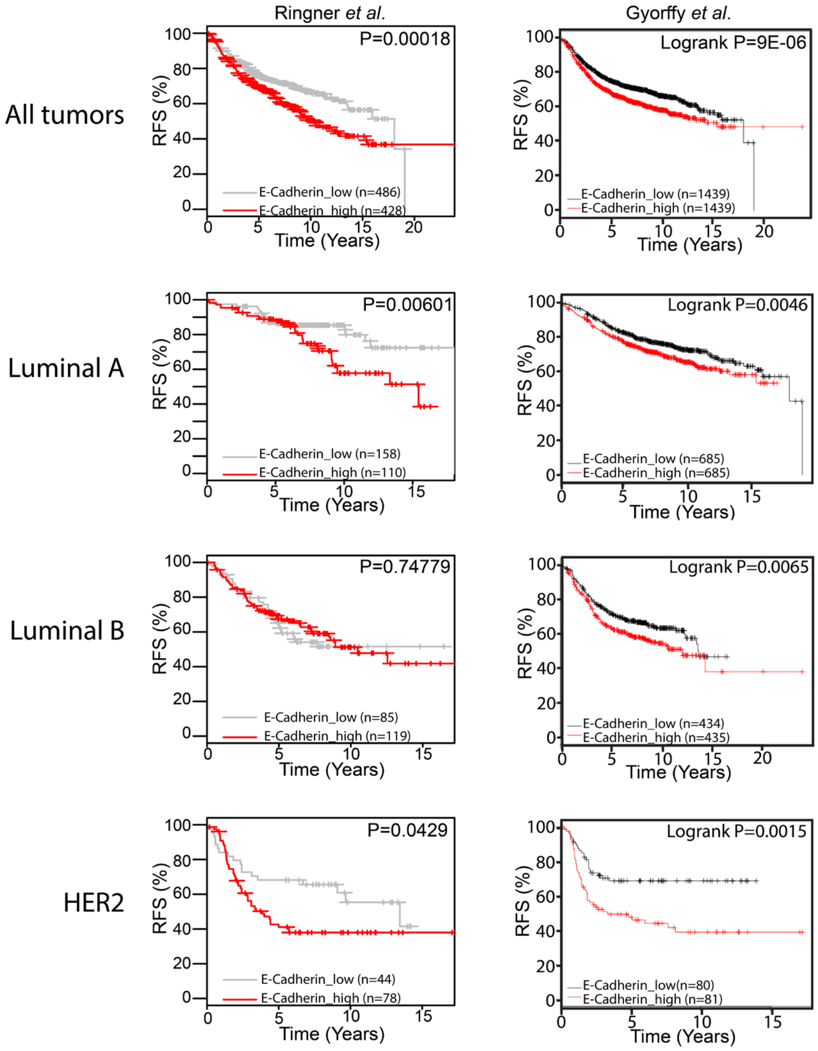

Sup Fig S1: Kaplan-Meier curves of E-Cadherin (201131_s_at) in 2 large public breast cancer database. Univariate analysis of unselect, luminal A, luminal B and HER2 tumors. PAM50 classification was used for the subtype classification of the Ringner *et al* dataset. Logrank P values are shown.

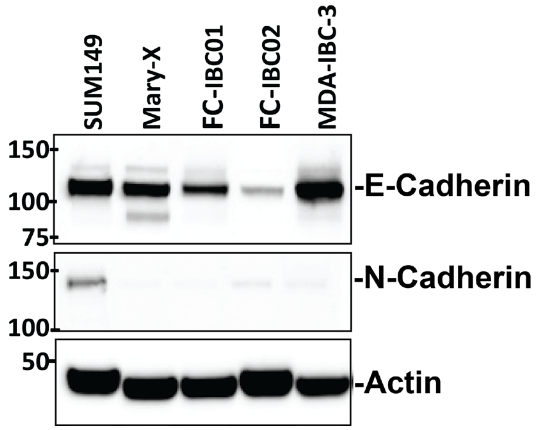

Sup Fig S2: Expression of E-Cadherin by 2 novel IBC xenograft cell lines. Western blot analysis of IBC cell lines including the novel xenograft line (FC-IBC01 and FC-IBC02) for E-Cadherin, N-Cadherin and actin.

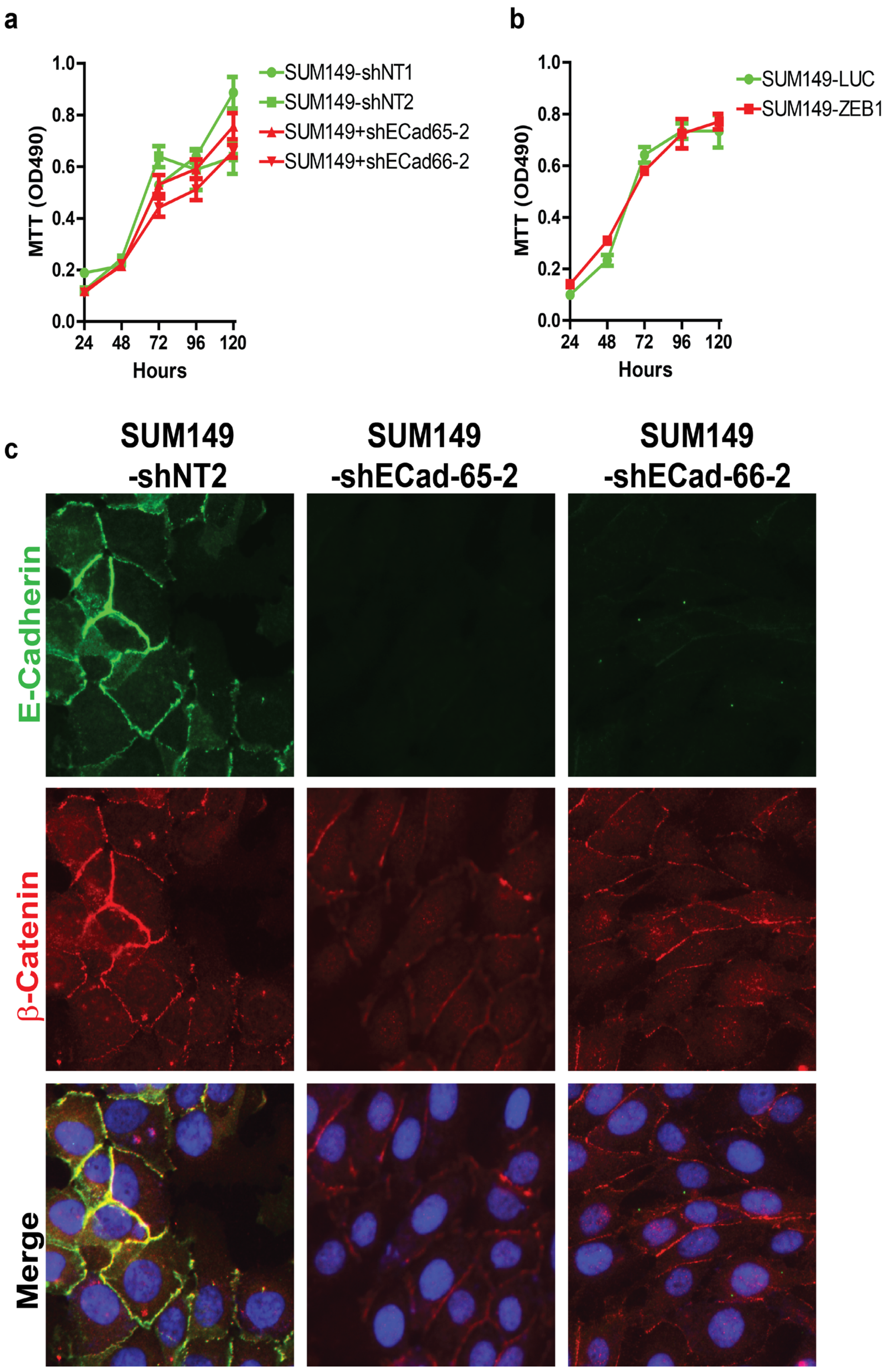

Sup Fig S3: (a) MTT growth measurements of SUM149-shNT(green) and SUM149-shECad clones (red). (b) MTT growth measurements of SUM149-LUC (green) and SUM149-ZEB1 (red). (c) Immunocytochemistry staining of E-Cadherin and beta-catenin in SUM149-shECad clones. Indirect dual immunofluorescence staining of E-Cadherin and beta-Catenin on SUM149-shNT and 2 E-Cadherin knockdown clone s (SUM149-shECad65 and SUM149-shECad). Reduced staining of E-Cadherin and beta-Catenin was observed in SUM149-
