# Supplementary material for: The Paradox of E-Cadherin: Role in response to hypoxia in the tumor microenvironment and regulation of energy metabolism
Source: Oncotarget. 2013 Mar 21;4(3):446–62. doi: 10.18632/oncotarget.872 (PMC3717307; doi:10.18632/oncotarget.872)
Supplement: Supplementary file 2 [file oncotarget-04-446-s002.docx]

The Paradox of E-Cadherin: Role in response to hypoxia in the tumor microenvironment and regulation of energy metabolism – Chu et al

**Sup. Table 1**: **List of differentially expressed in epithelial and mesenchymal SUM149 clones in vitro.**

Genes enriched in Epithelial SUM149 clones (>2xFold, FDR<0.397%)

Gene ID Gene Name Fold Change

205916_at S100A7 40.0

217963_s_at NGFRAP1 20.6

214146_s_at PPBP 13.8

202554_s_at GSTM3 13.7

202411_at IFI27 9.9

213478_at KIAA1026 9.2

215444_s_at TRIM31 8.3

208170_s_at TRIM31 7.9

209616_s_at CES1 7.2

202917_s_at S100A8 6.1

203535_at S100A9 6.1

227769_at GPR27 6.1

201911_s_at FARP1 5.9

214957_at ACTL8 5.8

224367_at BEX2 5.6

220138_at HAND1 5.5

218966_at MYO5C 5.5

227182_at SUSD3 5.2

214749_s_at ARMCX6 5.1

201061_s_at STOM 5.0

208006_at FOXI1 4.8

214079_at DHRS2 4.8

201650_at KRT19 4.7

228010_at PPP2R2C 4.4

223253_at EPDR1 4.3

213005_s_at ANKRD15 4.1

222830_at GRHL1 4.1

209949_at NCF2 4.0

205220_at GPR109B 3.9

209696_at FBP1 3.9

206714_at ALOX15B 3.9

1553613_s_at FOXC1 3.8

204614_at SERPINB2 3.8

1553973_a_at SPINK6 3.7

203509_at SORL1 3.7

231778_at DLX3 3.7

205691_at SYNGR3 3.6

201060_x_at STOM 3.6

219497_s_at BCL11A 3.5

220414_at CALML5 3.5

219127_at ATAD4 3.5

227307_at TSPAN18 3.4

200923_at LGALS3BP 3.4

213664_at SLC1A1 3.4

218454_at FLJ22662 3.4

206729_at TNFRSF8 3.4

225373_at C10orf54 3.3

210058_at MAPK13 3.3

205319_at PSCA 3.3

203238_s_at NOTCH3 3.3

206319_s_at SPINLW1 3.2

211906_s_at SERPINB4 3.2

219554_at RHCG 3.2

203477_at COL15A1 3.2

243871_at LOC644241 3.1

204735_at PDE4A 3.1

206067_s_at WT1 3.1

1554539_a_at RHOF 3.1

203108_at GPRC5A 3.1

211113_s_at ABCG1 3.1

205751_at SH3GL2 3.0

209291_at ID4 3.0

228819_at TSPAN18 3.0

204465_s_at INA 3.0

228359_at STS-1 3.0

229144_at KIAA1026 3.0

217975_at WBP5 3.0

209122_at ADFP 3.0

222891_s_at BCL11A 2.9

39248_at AQP3 2.9

203571_s_at C10orf116 2.9

219722_s_at GDPD3 2.9

203173_s_at C16orf62 2.9

206191_at ENTPD3 2.9

205501_at PDE10A 2.9

235252_at KSR1 2.9

205560_at PCSK5 2.8

1555416_a_at ALOX15B 2.8

201910_at FARP1 2.8

222746_s_at BSPRY 2.8

Gene ID Gene Name Fold Change 238686_at FBXO3 2.7

226039_at MGAT4A 2.8

210159_s_at TRIM31 2.8

224516_s_at CXXC5 2.7

222812_s_at RHOF 2.7

232318_s_at LINC00284 2.7

212560_at C11orf32 2.7

221805_at NEFL 2.7

210059_s_at MAPK13 2.7

223574_x_at PPP2R2C 2.7

219616_at FLJ21963 2.7

233955_x_at CXXC5 2.7

222773_s_at GALNT12 2.6

202510_s_at TNFAIP2 2.6

223454_at CXCL16 2.6

204542_at ST6GALNAC2 2.6

205306_x_at KMO 2.6

214370_at S100A8 2.6

209160_at AKR1C3 2.6

205559_s_at PCSK5 2.6

1553430_a_at EDARADD 2.6

201787_at FBLN1 2.6

222996_s_at CXXC5 2.6

229125_at ANKRD38 2.5

1552767_a_at HS6ST2 2.5

210347_s_at BCL11A 2.5

224189_x_at EHF 2.5

225822_at TMEM125 2.5

211138_s_at KMO 2.5

235563_at GPRC5A 2.5

222802_at EDN1 2.5

227396_at PTPRJ 2.5

215101_s_at CXCL5 2.5

206122_at SOX15 2.5

225941_at EIF4E3 2.5

228221_at SLC44A3 2.5

218072_at COMMD9 2.5

219710_at SH3TC2 2.5

218885_s_at GALNT12 2.5

1555787_at C11orf63 2.5

235467_s_at KCNC4 2.5

206463_s_at DHRS2 2.5

229432_at NAGS 2.4

210413_x_at SERPINB4 2.4

218432_at FBXO3 2.4

207113_s_at TNF 2.4

1558687_a_at FOXN1 2.4

201131_s_at CDH1 2.4

202260_s_at STXBP1 2.4

232361_s_at EHF 2.4

226582_at LOC400043 2.4

1553986_at RASEF 2.4

227641_at FBXL16 2.4

232195_at GPR158 2.4

1552946_at ZNF114 2.4

220795_s_at KIAA1446 2.4

205225_at ESR1 2.4

212444_at GPRC5A 2.4

219045_at RHOF 2.4

205518_s_at CMAH 2.3

219344_at SLC29A3 2.3

204063_s_at ULK2 2.3

205073_at CYP2J2 2.3

234317_s_at STOX2 2.3

203829_at ELP4 2.3

216953_s_at WT1 2.3

210999_s_at GRB10 2.3

230030_at HS6ST2 2.3

204724_s_at COL9A3 2.3

227264_at TRAF6 2.3

210571_s_at CMAH 2.3

204062_s_at ULK2 2.3

228027_at GPRASP2 2.3

202893_at UNC13B 2.3

217422_s_at CD22 2.3

223895_s_at EPN3 2.3

228072_at SYT12 2.3

227385_at PPAPDC2 2.3

224819_at TCEAL8 2.3

214240_at GAL 2.3

Gene ID Gene Name Fold Change 212062_at ATP9A 2.2

209911_x_at HIST1H2BD 2.2

200983_x_at CD59 2.2

222240_s_at ISYNA1 2.2

228302_x_at CAMK2N1 2.2

41469_at PI3 2.2

212415_at SEPT6 2.2

1557638_at BC040975 2.2

229534_at ACOT4 2.2

205681_at BCL2A1 2.2

236826_at TTC39B 2.2

1564307_a_at A2ML1 2.2

207850_at CXCL3 2.2

215071_s_at HIST1H2AC 2.2

217995_at SQRDL 2.2

36545_s_at SFI1 2.2

211340_s_at MCAM 2.2

214088_s_at FUT3 2.2

206002_at GPR64 2.2

223427_s_at EPB41L4B 2.2

225940_at EIF4E3 2.2

235583_at ILDR1 2.2

214651_s_at HOXA9 2.2

211922_s_at CAT 2.2

223500_at CPLX1 2.1

222383_s_at ALOXE3 2.1

211002_s_at TRIM29 2.1

226863_at FAM110C 2.1

234307_s_at KIF26A 2.1

201432_at CAT 2.1

210827_s_at ELF3 2.1

200984_s_at CD59 2.1

225645_at EHF 2.1

235144_at RASEF 2.1

1568593_a_at NUDT16P 2.1

224435_at C10orf58 2.1

Gene ID Gene Name Fold Change 200985_s_at CD59 2.1

213823_at HOXA11 2.1

204249_s_at LMO2 2.1

216905_s_at ST14 2.1

203947_at CSTF3 2.1

217185_s_at ZNF259 2.1

202504_at TRIM29 2.1

239435_x_at APXL2 2.1

238513_at PRRG4 2.1

219825_at CYP26B1 2.1

205660_at OASL 2.1

219860_at LY6G5C 2.1

207291_at PRRG4 2.1

214971_s_at ST6GAL1 2.1

220161_s_at EPB41L4B 2.1

209410_s_at GRB10 2.1

219680_at NOD9 2.1

202005_at ST14 2.1

203528_at SEMA4D 2.1

220318_at EPN3 2.1

202357_s_at CFB 2.1

203691_at PI3 2.0

209293_x_at ID4 2.0

204567_s_at ABCG1 2.0

221656_s_at ARHGEF10L 2.0

223732_at SLC23A1 2.0

214290_s_at HIST2H2AA3 2.0

209083_at CORO1A 2.0

232290_at GPRC5A 2.0

217884_at NAT10 2.0

226988_s_at MYH14 2.0

212463_at CD59 2.0

1569555_at GDA 2.0

201009_s_at TXNIP 2.0

218928_s_at SLC37A1 2.0

232054_at PCDH20 2.0

Genes enriched in Mesenchymal SUM149 clones in vitro (>2xFold, FDR<0.397%)

Gene ID Gene Name Fold Change

218468_s_at GREM1 12.5

218469_at GREM1 11.7

209533_s_at PLAA 8.9

213948_x_at IGSF4B 7.6

201426_s_at VIM 7.4

221541_at CRISPLD2 7.0

226844_at MOBKL2B 6.7

222686_s_at FLJ11151 5.8

203887_s_at THBD 5.6

210119_at KCNJ15 5.2

203889_at SCG5 5.2

208791_at CLU 5.0

225919_s_at C9orf72 5.0

202391_at BASP1 4.9

203888_at THBD 4.9

212233_at MAP1B 4.8

211806_s_at KCNJ15 4.6

221731_x_at CSPG2 4.4

1553133_at C9orf72 4.4

211919_s_at CXCR4 4.1

235548_at APCDD1L 4.0

205097_at SLC26A2 4.0

209201_x_at CXCR4 4.0

217028_at CXCR4 4.0

204620_s_at CSPG2 3.9

236532_at C11orf87 3.9

219276_x_at C9orf82 3.8

203434_s_at MME 3.8

209301_at CA2 3.7

203440_at CDH2 3.7

202729_s_at LTBP1 3.6

224963_at SLC26A2 3.6

201976_s_at MYO10 3.5

210959_s_at SRD5A1 3.5

224959_at SLC26A2 3.4

212488_at COL5A1 3.4

204519_s_at PLLP 3.4

229347_at LOC100505738 3.4

203789_s_at SEMA3C 3.3

203435_s_at MME 3.2

201095_at DAP 3.2

219174_at IFT74 3.2

204675_at SRD5A1 3.2

211056_s_at SRD5A1 3.2

208792_s_at CLU 3.0

211896_s_at DCN 3.0

206623_at PDE6A 3.0

238751_at SORBS2 3.0

201843_s_at EFEMP1 3.0

203729_at EMP3 2.9

211813_x_at DCN 2.9

204115_at GNG11 2.9

201737_s_at MARCH6 2.9

226237_at COL8A1 2.9

223020_at CLPTM1L 2.9

209654_at KIAA0947 2.9

211571_s_at CSPG2 2.8

228737_at C20orf100 2.8

203441_s_at CDH2 28

227846_at GPR176 2.8

229641_at CCBE1 2.8

201069_at MMP2 2.7

215646_s_at CSPG2 2.7

229268_at FAM105B 2.7

219200_at FASTKD3 2.7

232231_at RUNX2 2.7

202466_at POLS 2.7

203200_s_at MTRR 2.7

224767_at RPL37 2.6

211677_x_at IGSF4B 2.6

221921_s_at IGSF4B 2.6

213338_at TMEM158 2.6

224763_at RPL37 2.6

211596_s_at LRIG1 2.6

201842_s_at EFEMP1 2.6

210764_s_at CYR61 2.6

208178_x_at TRIO 2.6

209011_at TRIO 2.6

212498_at AF056433 2.6

206969_at KRT34 2.5

202686_s_at AXL 2.5

Gene ID Gene Name Fold Change

223092_at ANKH 2.5

202766_s_at FBN1 2.5

201818_at AYTL2 2.5

223247_at MED10 2.5

209013_x_at TRIO 2.5

202728_s_at LTBP1 2.5

210261_at KCNK2 2.5

201736_s_at MARCH6 2.5

208763_s_at TSC22D3 2.4

239169_at RDM1 2.4

226935_s_at CLPTM1L 2.4

235705_at TRIO 2.4

226084_at MAP1B 2.4

203413_at NELL2 2.4

202207_at ARL4C 2.4

219182_at CHST5 2.4

241763_s_at FBXO32 2.4

224331_s_at MRPL36 2.4

1554026_a_at MYO10 2.4

205239_at AREG 2.4

209114_at TSPAN1 2.4

226875_at DOCK11 2.4

207001_x_at TSC22D3 2.4

243805_at CCBE1 2.4

1559960_x_at SYCE1L 2.4

223076_s_at NSUN2 2.4

225670_at LOC134145 2.3

202052_s_at RAI14 2.3

202558_s_at STCH 2.3

225803_at FBXO32 2.3

206033_s_at DSC3 2.3

230143_at RNF165 2.3

229831_at CNTN3 2.3

203606_at NDUFS6 2.3

204686_at IRS1 2.3

209335_at DCN 2.3

203625_x_at SKP2 2.2

208696_at CCT5 2.2

203199_s_at MTRR 2.2

212256_at GALNT10 2.2

201289_at CYR61 2.2

239135_at CPPED1 2.2

210567_s_at SKP2 2.2

218718_at PDGFC 2.2

223094_s_at ANKH 2.2

218665_at FZD4 2.2

222719_s_at PDGFC 2.2

243707_at Hs.145520 2.2

204011_at SPRY2 2.2

202685_s_at AXL 2.1

209010_s_at TRIO 2.1

233446_at ONECUT2 2.1

1552660_a_at C5orf22 2.1

204140_at TPST1 2.1

201042_at TGM2 2.1

225173_at ARHGAP18 2.1

220698_at MGC4294 2.1

1555007_s_at WDR66 2.1

64900_at TMEM231 2.1

204784_s_at MLF1 2.1

200602_at APP 2.1

201506_at TGFBI 2.1

204033_at TRIP13 2.1

219789_at NPR3 2.1

227764_at LYPD6 2.1

1558930_at LINC00460 2.1

235134_at BC007851 2.1

205110_s_at FGF13 2.1

225152_at ZNF622 2.0

236075_s_at ZNF169 2.0

235990_at POLD4 2.0

217989_at DHRS8 2.0

206702_at TEK 2.0

203917_at CXADR 2.0

213262_at SACS 2.0

219265_at MOBKL2B 2.0

231879_at COL12A1 2.0

211042_x_at MCAM 2.0

224870_at KIAA0114 2.0

211980_at COL4A1 2.0

**Sup. Table 2**: List of differentially expressed in epithelial and mesenchymal SUM149 clones in vivo

Genes enriched in Epithelial SUM149 clones in vivo (>2xFold, FDR<0.0%)

Gene ID Gene Name Fold Change

204475_at MMP1 40.7

205476_at CCL20 16.7

238047_at ARHGAP36 15.0

205067_at IL1B 13.7

229802_at WISP1 13.6

202859_x_at IL8 12.8

202238_s_at NNMT 11.0

1554997_a_at PTGS2 10.7

202237_at NNMT 10.3

220322_at IL1F9 10.3

204748_at PTGS2 10.3

221872_at RARRES1 9.8

206392_s_at RARRES1 9.0

206391_at RARRES1 8.6

235821_at WISP1 8.5

217757_at A2M 8.2

39402_at IL1B 8.2

211506_s_at IL8 7.8

213060_s_at CHI3L2 7.7

229152_at FDCSP 7.6

223541_at HAS3 7.5

213524_s_at G0S2 7.4

206796_at WISP1 7.3

205681_at BCL2A1 7.2

207850_at CXCL3 7.0

202638_s_at ICAM1 6.9

229230_at OSTα 6.7

219795_at SLC6A14 6.6

209122_at ADFP 6.6

206569_at IL24 6.2

202357_s_at CFB 6.1

205767_at EREG 5.8

214749_s_at ARMCX6 5.7

217767_at C3 5.6

204470_at CXCL1 5.6

203562_at FEZ1 5.6

230720_at RNF182 5.6

219523_s_at ODZ3 5.6

210118_s_at IL1A 5.5

217858_s_at ARMCX3 5.5

205207_at IL6 5.5

211312_s_at WISP1 5.3

1569583_at EREG 5.3

230251_at LINC00473 5.3

202637_s_at ICAM1 5.3

227697_at SOCS3 5.1

214957_at ACTL8 5.0

209396_s_at CHI3L1 5.0

209395_at CHI3L1 5.0

210423_s_at SLC11A1 4.8

209616_s_at CES1 4.8

204014_at DUSP4 4.8

209656_s_at TMEM47 4.5

227769_at GPR27 4.4

226034_at DUSP4 4.4

1554833_at MCTP2 4.3

225842_at PHLDA1 4.2

209774_x_at CXCL2 4.2

227961_at CTSB 4.2

212014_x_at CD44 4.1

209835_x_at CD44 4.1

1568920_at SOX5 4.1

204933_s_at TNFRSF11B 4.1

226804_at FAM20A 4.1

212531_at LCN2 4.1

239893_at MCTP2 4.0

200986_at SERPING1 4.0

218810_at ZC3H12A 3.9

215223_s_at SOD2 3.9

210102_at LOH11CR2A 3.8

203477_at COL15A1 3.8

210916_s_at CD44 3.8

208451_s_at C4A 3.8

229221_at CD44 3.8

205445_at PRL 3.8

226834_at Hs.504187 3.7

221009_s_at ANGPTL4 3.7

210357_s_at SMOX 3.7

1557905_s_at CD44 3.7

209641_s_at ABCC3 3.6

1559910_at Hs.655648 3.6

1555486_a_at PRR5L 3.6

Gene ID Gene Name Fold Change

220603_s_at MCTP2 3.6

204490_s_at CD44 3.6

201389_at ITGA5 3.6

202643_s_at TNFAIP3 3.6

204015_s_at DUSP4 3.5

200878_at EPAS1 3.5

232113_at AK09579 3.5

219938_s_at PSTPIP2 3.5

202644_s_at TNFAIP3 3.5

224657_at ERRFI1 3.5

210367_s_at PTGES 3.5

236599_at SERPINE2 3.4

243109_at MCTP2 3.4

202510_s_at TNFAIP2 3.4

228082_at ASAM 3.4

1555680_a_at SMOX 3.4

204489_s_at CD44 3.3

227432_s_at ZNF557 3.3

205842_s_at JAK2 3.3

205304_s_at KCNJ8 3.3

227307_at TSPAN18 3.3

209930_s_at NFE2 3.2

230563_at RASGEF1A 3.2

233177_s_at PNKD 3.2

213275_x_at CTSB 3.2

228846_at MXD1 3.2

221489_s_at SPRY4 3.1

226858_at CSNK1E 3.1

202897_at SIRPA 3.1

233388_at CA12 3.1

223333_s_at ANGPTL4 3.1

202016_at MEST 3.1

205042_at GNE 3.1

212063_at CD44 3.1

205011_at VWA5A 3.1

222529_at SLC25A37 3.1

36711_at MAFF 3.1

242271_at SLC26A9 3.1

220655_at TNIP3 3.1

205997_at ADAM28 3.0

1559117_at AK091983 3.0

214428_x_at C4A 3.0

228758_at BCL6 3.0

230682_x_at ABCC3 3.0

218332_at BEX1 3.0

243296_at PBEF1 3.0

228523_at NANOS1 3.0

205943_at TDO2 3.0

205513_at TCN1 3.0

224209_s_at GDA 3.0

201041_s_at DUSP1 3.0

207695_s_at IGSF1 2.9

201743_at CD14 2.9

228819_at TSPAN18 2.9

235419_at ERRFI1 2.9

219522_at FJX1 2.9

204924_at TLR2 2.9

236826_at TTC39B 2.8

201631_s_at IER3 2.8

1559663_at ACSL4 2.8

220133_at ODAM 2.8

208161_s_at ABCC3 2.8

207388_s_at PTGES 2.8

223217_s_at NFKBIZ 2.8

202554_s_at GSTM3 2.8

206584_at LY96 2.8

220795_s_at KIAA1446 2.8

221081_s_at DENND2D 2.8

226275_at MXD1 2.8

209696_at FBP1 2.8

219179_at DACT1 2.7

225283_at ARRDC4 2.7

231559_at NNMT 2.7

229933_at C1orf74 2.7

215990_s_at BCL6 2.7

222528_s_at SLC25A37 2.7

225381_at MIR100HG 2.7

217523_at CD44 2.7

214719_at SLC46A3 2.7

1559759_at KIFC3 2.7

228570_at BTBD11 2.7

209732_at CLEC2B 2.7

Gene ID Gene Name Fold Change

211840_s_at PDE4D 2.7

225973_at TAP2 2.7

206058_at SLC6A12 2.7

209765_at ADAM19 2.7

224367_at BEX2 2.7

203504_s_at ABCA1 2.7

1565868_at CD44 2.6

211573_x_at TGM2 2.6

220407_s_at TGFB2 2.6

204932_at TNFRSF11B 2.6

213664_at SLC1A1 2.6

236161_at Hs.559488 2.6

241418_at LOC344887 2.6

236610_at PDE4D 2.6

219423_x_at TNFRSF25 2.6

204836_at GLDC 2.6

221773_at ELK3 2.6

219383_at PRR5L 2.6

213927_at MAP3K9 2.6

226206_at MAFK 2.6

201621_at NBL1 2.6

202626_s_at LYN 2.6

219926_at POPDC3 2.6

214696_at MIR22HG 2.6

241359_at TLCD2 2.6

242275_at AI589190 2.6

231932_at TRAF3IP3 2.5

204908_s_at BCL3 2.5

226542_at Hs.547576 2.5

209655_s_at TMEM47 2.5

215001_s_at GLUL 2.5

229160_at MUM1L1 2.5

235751_s_at VMO1 2.5

210754_s_at LYN 2.5

222444_at ARMCX3 2.4

212190_at SERPINE2 2.4

225316_at MFSD2 2.4

201925_s_at CD55 2.4

212110_at SLC39A14 2.4

205443_at SNAPC1 2.4

1569962_at Hs.680771 2.4

224583_at COTL1 2.4

230240_at DYRK3 2.4

229854_at OBSCN 2.4

230711_at EPAS1 2.4

218309_at CAMK2N1 2.4

239296_at ZFHX1B 2.4

223741_s_at TTYH2 2.4

210663_s_at KYNU 2.4

227385_at PPAPDC2 2.4

1564307_a_at A2ML1 2.4

243439_at ZNF418 2.3

217497_at ECGF1 2.3

221059_s_at COTL1 2.3

200648_s_at GLUL 2.3

228302_x_at CAMK2N1 2.3

221477_s_at SOD2 2.3

227396_at PTPRJ 2.3

242868_at EPAS1 2.3

229021_at MCTP2 2.3

223659_at TMPRSS13 2.3

214438_at HLX1 2.3

211302_s_at PDE4B 2.3

241763_s_at FBXO32 2.3

220177_s_at TMPRSS3 2.3

229438_at FAM20C 2.3

205319_at PSCA 2.3

226582_at LOC400043 2.3

218611_at IER5 2.3

214211_at FTH1 2.3

202834_at AGT 2.2

225251_at RAB24 2.2

Gene ID Gene Name Fold Change

1556499_s_at COL1A1 2.2

226498_at FLT1 2.2

205582_s_at GGTLA1 2.2

229144_at KAZN 2.2

204166_at SBNO2 2.2

224655_at AK3 2.2

205599_at TRAF1 2.2

228528_at MIR29B2/C 2.2

203710_at ITPR1 2.2

223218_s_at NFKBIZ 2.2

204592_at DLG4 2.2

233364_s_at AK021804 2.2

204491_at PDE4D 2.2

1559322_at PTP4A1 2.2

232882_at FOXO1A 2.2

205137_x_at USH1C 2.2

227970_at GPR157 2.2

207978_s_at NR4A3 2.2

206940_s_at POU4F1 2.2

221173_at USH1C 2.2

239798_at PDK1 2.2

1558299_at SIRPA 2.2

212463_at CD59 2.1

207113_s_at TNF 2.1

210836_x_at PDE4D 2.1

222666_s_at RCL1 2.1

223059_s_at FAM107B 2.1

209959_at NR4A3 2.1

200629_at WARS 2.1

204639_at ADA 2.1

203708_at PDE4B 2.1

214791_at SP140L 2.1

227183_at MIR143HG 2.1

211323_s_at ITPR1 2.1

225387_at TSPAN5 2.1

217185_s_at ZNF259 2.1

209890_at TSPAN5 2.1

204385_at KYNU 2.1

242874_at ENSG00000260711 2.1

204621_s_at NR4A2 2.1

203879_at PIK3CD 2.1

203505_at ABCA1 2.1

205945_at IL6R 2.1

214340_at ALOX12P2 2.1

201482_at QSCN6 2.1

211282_x_at TNFRSF25 2.1

200808_s_at ZYX 2.1

238490_at KIAA2026 2.1

212419_at ZCCHC24 2.1

227467_at RDH10 2.1

237459_at PCTK2 2.1

232750_at TNS1 2.0

238135_at AGTRAP 2.0

200054_at ZNF259 2.0

221920_s_at SLC25A37 2.0

229432_at NAGS 2.0

205282_at LRP8 2.0

218136_s_at SLC25A37 2.0

238686_at FBXO3 2.0

229522_at HSPC105 2.0

200628_s_at WARS 2.0

204735_at PDE4A 2.0

217388_s_at KYNU 2.0

227188_at C21orf63 2.0

224151_s_at AK3 2.0

202862_at FAH 2.0

217202_s_at GLUL 2.0

222961_at AF116607 2.0

219367_s_at NRP2 2.0

242997_at AW664311 2.0

211665_s_at SOS2 2.0

213792_s_at INSR 2.0

Gene enriched in mesenchymal SUM149 clones in vivo (>2xFold, FDR=0.0%)

Gene ID Gene Name Fold Change

225275_at EDIL3 16.3

229357_at ADAMTS5 15.3

219935_at ADAMTS5 13.3

209533_s_at PLAA 9.5

235368_at ADAMTS5 8.4

1559942_at MDFIC 5.5

219276_x_at C9orf82 5.2

219174_at IFT74 5.2

208998_at UCP2 5.2

222686_s_at FLJ11151 4.7

209581_at HRASLS3 4.6

220354_at MCF2L-AS1 4.4

218510_x_at FAM134B 4.3

226534_at KITLG 4.1

227863_at CTSD 4.1

232481_s_at SLITRK6 4.1

223092_at ANKH 4.0

202391_at BASP1 4.0

213880_at LGR5 4.0

35147_at MCF2L 3.9

205358_at GRIA2 3.8

213240_s_at KRT4 3.8

201976_s_at MYO10 3.8

226844_at MOBKL2B 3.8

225919_s_at C9orf72 3.7

209569_x_at D4S234E 3.6

229347_at LOC100505738 3.6

212935_at MCF2L 3.5

218804_at TMEM16A 3.4

201095_at DAP 3.4

218532_s_at FLJ20152 3.4

235134_at BC007851 3.4

233606_at BMP5 3.4

209654_at KIAA0947 3.3

219790_s_at NPR3 3.3

203824_at TSPAN8 3.3

239135_at CPPED1 3.3

204452_s_at FZD1 3.3

207173_x_at CDH11 3.2

232176_at SLITRK6 3.2

212233_at MAP1B 3.2

230472_at IRX1 3.2

223094_s_at ANKH 3.2

243531_at ORAOV1 3.2

206103_at RAC3 3.1

206023_at NMU 3.1

201565_s_at ID2 3.1

1554696_s_at TYMS 3.0

227209_at CNTN1 3.0

221521_s_at GINS2 3.0

1555269_a_at TMEM16A 3.0

207935_s_at KRT13 2.9

203130_s_at KIF5C 2.9

229715_at ENSG00000260196 2.9

223020_at CLPTM1L 2.9

212498_at AF056433 2.9

225670_at FAM173B 2.9

202780_at OXCT1 2.9

201737_s_at MARCH6 2.9

1564573_at IFITM10 2.9

219528_s_at BCL11B 2.8

207172_s_at CDH11 2.8

225232_at MTMR12 2.8

227985_at LOC100506098 2.8

1554026_a_at MYO10 2.8

204162_at KNTC2 2.8

218201_at NDUFB2 2.8

212387_at TCF4 2.8

229470_at FAM105B 2.7

226781_at HSPC268 2.7

1553778_at WBSCR27 2.7

200602_at APP 2.7

212382_at TCF4 2.7

226780_s_at HSPC268 2.7

203953_s_at CLDN3 2.7

229442_at C18orf54 2.7

205047_s_at ASNS 2.7

213301_x_at TRIM24 2.6

1553133_at C9orf72 2.6

204675_at SRD5A1 2.6

228049_x_at LOC100507303 2.6

235182_at C20orf82 2.6

231195_at FLJ44186 2.6

Gene ID Gene Name Fold Change

206737_at WNT11 2.6

223709_s_at WNT10A 2.6

242127_at IL28RA 2.6

228323_at CASC5 2.6

1554867_a_at PRR16 2.6

1557128_at FAM111B 2.6

229268_at FAM105B 2.6

204218_at C11orf51 2.6

208696_at CCT5 2.6

222895_s_at BCL11B 2.6

212385_at TCF4 2.6

206140_at LHX2 2.6

218200_s_at NDUFB2 2.6

212154_at SDC2 2.6

205070_at ING3 2.6

210253_at HTATIP2 2.5

242138_at DLX1 2.5

204602_at DKK1 2.5

212386_at TCF4 2.5

219188_s_at LRP16 2.5

234980_at TMEM56 2.5

203606_at NDUFS6 2.5

1556806_at DAP 2.5

202589_at TYMS 2.5

212157_at SDC2 2.5

210959_s_at SRD5A1 2.5

227197_at SGEF 2.5

203805_s_at FANCA 2.5

32137_at JAG2 2.5

203625_x_at SKP2 2.5

226125_at SLC9A3 2.5

201341_at ENC1 2.5

213467_at RND2 2.4

222146_s_at TCF4 2.4

212345_s_at CREB3L2 2.4

232134_at POLS 2.4

228124_at ABHD12 2.4

211056_s_at SRD5A1 2.4

211675_s_at MDFIC 2.4

226935_s_at CLPTM1L 2.4

224587_at SUB1 2.4

1552470_a_at ABHD11 2.4

238273_at SLC13A4 2.4

223274_at TCF19 2.4

202295_s_at CTSH 2.4

202580_x_at FOXM1 2.4

213707_s_at DLX5 2.4

235148_at KRTCAP3 2.4

1558487_a_at TMED4 2.4

219306_at KIF15 2.4

228399_at OSR1 2.4

224331_s_at MRPL36 2.4

213248_at LOC221362 2.4

226084_at MAP1B 2.4

204460_s_at RAD1 2.4

237304_at SYCE2 2.4

219389_at SUSD4 2.3

209784_s_at JAG2 2.3

204451_at FZD1 2.3

220953_s_at MTMR12 2.3

204353_s_at POT1 2.3

236947_at SEMA3C 2.3

205430_at BMP5 2.3

221727_at SUB1 2.3

1556344_at LOC150051 2.3

207147_at DLX2 2.3

204359_at FLRT2 2.3

226109_at C21orf91 2.3

203753_at TCF4 2.3

204444_at KIF11 2.3

238724_at BPGM 2.3

201818_at AYTL2 2.3

219200_at FASTKD3 2.3

235705_at TRIO 2.3

229444_at LOC100131607 2.3

228271_at SND1 2.3

221591_s_at FAM64A 2.3

228110_x_at Hs.530053 2.3

226779_at LMBRD2 2.3

239449_at ANKH 2.2

206927_s_at GUCY1A2 2.2

235253_at RAD1 2.2

235967_at LOC100127891 2.2

Gene ID Gene Name Fold Change

203696_s_at RFC2 2.2

214804_at CENPI 2.2

203413_at NELL2 2.2

214845_s_at CALU 2.2

206632_s_at APOBEC3B 2.2

204033_at TRIP13 2.2

221893_s_at ADCK2 2.2

204798_at MYB 2.2

210983_s_at MCM7 2.2

201736_s_at MARCH6 2.2

225458_at PP7080 2.2

211219_s_at LHX2 2.2

214512_s_at SUB1 2.2

227085_at H2AFV 2.2

1559960_x_at SYCE1L 2.2

1568592_at RNF36 2.2

233999_s_at TTC26 2.2

230591_at LOC729887 2.2

219531_at CEP72 2.2

201764_at TMEM106C 2.2

205066_s_at ENPP1 2.2

227502_at RDX 2.2

201830_s_at NET1 2.2

219342_at CASD1 2.2

210216_x_at RAD1 2.2

227313_at MGC40499 2.2

235282_at SP2 2.2

211042_x_at MCAM 2.2

202466_at POLS 2.2

209172_s_at CENPF 2.2

240815_at SEMA3C 2.2

218558_s_at MRPL39 2.2

203853_s_at GAB2 2.2

225355_at NEURL1B 2.2

213891_s_at TCF4 2.1

212483_at NIPBL 2.1

228033_at E2F7 2.1

202148_s_at PYCR1 2.1

204159_at CDKN2C 2.1

239169_at RDM1 2.1

44120_at ADCK2 2.1

217803_at GOLPH3 2.1

213599_at OIP5 2.1

203626_s_at SKP2 2.1

226504_at FAM109B 2.1

218654_s_at MRPS33 2.1

204354_at POT1 2.1

1554079_at GALNTL4 2.1

Gene ID Gene Name Fold Change

209714_s_at CDKN3 2.1

224586_x_at SUB1 2.1

235219_at C5orf55 2.1

226031_at CCDC132 2.1

223247_at MED10 2.1

223165_s_at IHPK2 2.1

228382_at FAM105B 2.1

223457_at COPG2 2.1

213411_at ADAM22 2.1

223076_s_at NSUN2 2.1

219785_s_at FBXO31 2.1

205206_at KAL1 2.1

202325_s_at ATP5J 2.1

1555758_a_at CDKN3 2.1

219555_s_at CENPN 2.1

219779_at ZFHX4 2.1

223493_at FBXO4 2.1

222742_s_at RABL5 2.1

230449_x_at ENSG00000253948 2.1

1568932_at ENSG00000260581 2.1

234726_s_at TMEM168 2.1

214734_at EXPH5 2.1

220419_s_at USP25 2.1

236546_at POLA2 2.1

207828_s_at CENPF 2.0

207761_s_at METTL7A 2.0

1053_at RFC2 2.0

238898_at LOC100505730 2.0

203675_at NUCB2 2.0

225152_at ZNF622 2.0

203789_s_at SEMA3C 2.0

219978_s_at NUSAP1 2.0

201829_at NET1 2.0

223622_s_at HYI 2.0

235545_at DEPDC1 2.0

212023_s_at MKI67 2.0

210567_s_at SKP2 2.0

224870_at DANCR 2.0

226041_at NAPE-PLD 2.0

204391_x_at TRIM24 2.0

219114_at C3orf18 2.0

200756_x_at CALU 2.0

210162_s_at NFATC1 2.0

210109_at NAG8 2.0

227771_at LIFR 2.0

230353_at MIR497HG 2.0

217842_at LUC7L2 2.0

214953_s_at APP 2.0

**Sup. Table 3:** List of cell line clones used in this study.

|  | Clone Name | Comments | Catalog (Source) |  |
| --- | --- | --- | --- | --- |
| 1 | SUM149-shNT1 | MISSION® pLKO.1-puro Non-Mammalian shRNA Control Plasmid DNA | SHC002 (Sigma) |  |
| 2 | SUM149-shNT2 | MISSION® pLKO.1-puro Non-Mammalian shRNA Control Plasmid DNA | SHC002 (Sigma) |  |
| 3 | SUM149-shECad65-1 | MISSION® E-Cadherin shRNA knockdown Plasmid single cell clone | TRCN0000039665 (Sigma) |  |
| 4 | SUM149-shECad65-2 | MISSION® E-Cadherin shRNA knockdown Plasmid single cell clone | TRCN0000039665 (Sigma) |  |
| 5 | SUM149-shECad66-1 | MISSION® E-Cadherin shRNA knockdown Plasmid single cell clone | TRCN0000039666 (Sigma) |  |
| 6 | SUM149-shECad65-2 | MISSION® E-Cadherin shRNA knockdown Plasmid single cell clone E-Cadherin knockdown single cell clone | TRCN0000039666 (Sigma) |  |
| 7 | SUM149-shECad65-1-LUC | +Luciferase (firefly)-2A-RFP (Bsd) | LVP009 (Gentarget) |  |
| 8 | SUM149-shECad65-2-LUC | +Luciferase (firefly)-2A-RFP (Bsd) | LVP009 (Gentarget) |  |
| 9 | SUM149-shECad66-1-LUC | +Luciferase (firefly)-2A-RFP (Bsd) | LVP009 (Gentarget) |  |
| 10 | SUM149-shECad65-2-LUC | +Luciferase (firefly)-2A-RFP (Bsd) | LVP009 (Gentarget) |  |
| 11 | SUM149-LUC-c3 | Vector single cell clone | pBMN-LUC-IRES-GFP (see Methods) |  |
| 12 | SUM149-LUC-c39 | Vector single cell clone | pBMN-LUC-IRES-GFP (see Methods) |  |
| 13 | SUM149-LUC-c42 | Vector single cell clone | pBMN-LUC-GFP (see Methods) |  |
| 14 | SUM149-ZEB1-c16 | ZEB1 overexpression single cell clone | pBMN-ZEB1-GFP (see Methods) |  |
| 15 | SUM149-ZEB1-c30 | ZEB1 overexpression single cell clone | pBMN-ZEB1-GFP (see Methods) |  |
| 16 | SUM149-ZEB1-c38 | ZEB1 overexpression single cell clone | pBMN-ZEB1-GFP (see Methods) |  |
| 17 | SUM149-LUC-c39-shNS-c4 | GIPZ Non-silencing Lentiviral shRNA Control single cell clone | RHS4346 (Open Biosystems) |  |
| 18 | SUM149-LUC-c39-shNS-c7 | GIPZ Non-silencing Lentiviral shRNA Control single cell clone | RHS4346 (Open Biosystems) |  |
| 19 | SUM149-LUC-c39-shHIF-1α-c6 | GIPZ Lentiviral Human HIF1α shRNA single cell clone | V2LHS_132150 (Open Biosystems) |  |
| 20 | SUM149-LUC-c39-shHIF-1 α -c19 | GIPZ Lentiviral Human HIF1α shRNA single cell clone | V3LHS_374856 (Open Biosystems) |  |
| 21 | SUM149-shECad65-2-LUC-iGFP | Control empty vector | pBMN-IRES-GFP (Addgene) |  |
| 22 | SUM149-shECad66-2-LUC -iGFP | Control empty vector | pBMN-IRES-GFP (Addgene) |  |
| 23 | SUM149-shECad65-2-LUC-HIF-1α | HIF-1α overexpression | pBMN-HIF-1α-IRES-GFP (see Methods) |  |
| 24 | SUM149-shECad66-2-LUC -HIF-1α | HIF-1α overexpression | pBMN-HIF-1α-IRES-GFP (see Methods) |  |
| 25 | 4T1-shNS14 | GIPZ Non-silencing Lentiviral shRNA Control single cell clone | RHS4346 (Open Biosystems)) |  |
| 26 | 4T1-shECad707-07 | GIPZ Lentiviral Mouse Cdh1 shRNA single cell clone | V2LMM_65707 (Open Biosystems) |  |
| 27 | 4T1-shECad306-47(A) | GIPZ Lentiviral Mouse Cdh1 shRNA single cell clone | V2LMM_65306 (Open Biosystems) |  |
| 28 | 4T1-shECad707-23(B) | GIPZ Lentiviral Mouse Cdh1 shRNA single cell clone | V2LMM_65707 (Open Biosystems) |  |
| 29 | MaryX+shNT-LiP | Modified MISSION® pLKO.1-puro plasmid with Luciferase gene | pLKO-NT-LiP (see Methods) |  |
| 30 | MaryX+shECad66-LiP | Modified MISSION® E-Cadherin shRNA knockdown Plasmid [TRCN0000039666 (Sigma)] with Luciferase gene | pLKO-shECad-LiP (see Methods) |  |
